# Fully 3D‐Printed Soft Capacitive Sensor of High Toughness and Large Measurement Range

**DOI:** 10.1002/advs.202410284

**Published:** 2025-01-07

**Authors:** Fei Xiao, Zhuoheng Wei, Zhipeng Xu, Hao Wang, Jisen Li, Jian Zhu

**Affiliations:** ^1^ School of Science and Engineering The Chinese University of Hong Kong Shenzhen 518172 P. R. China; ^2^ Soft Robotics Center Shenzhen Institute of Artificial Intelligence and Robotics for Society Shenzhen 518129 P. R. China

**Keywords:** 3D printing, fully 3D‐printed sensors and actuators, simultaneous sensing and actuation, soft robots, wearable devices

## Abstract

Soft capacitive sensors are widely utilized in wearable devices, flexible electronics, and soft robotics due to their high sensitivity. However, they may suffer delamination and/or debonding due to their low interfacial toughness. In addition, they usually exhibit a small measurement range resulting from their limited stiffness variation range. In this paper, soft silicone‐based capacitive sensors are developed by using a customized multimaterial 3D printer. By curing silicone materials simultaneously, the continuous conductive and dielectric layers achieve a substantial interfacial toughness of 1036 J·m^−2^. The sensor with tilted thin‐plate dielectrics exhibits interfacial toughness of 645 J·m^−2^ or 339 J·m^−2^ in the transverse or longitudinal direction, respectively. Additionally, the sensors demonstrate a broad measurement range from 0.85 Pa to 5000 kPa. This extended range is facilitated by the significant stiffness variation of the separated tilted thin‐plate dielectrics, ranging from 0.56 kPa to 19.76 MPa. Two applications of these fully printed soft sensors, including an intelligent sensorized insole and a robotic hand combining both soft actuators and soft sensors are showcased. It is believed that the strategy, employing 3D printing for soft microstructured sensors, is a general approach not only applicable for improving the performance of soft sensors, but also conducive to designing powerful soft functional devices.

## Graphical abstract

This study develops soft silicone‐based capacitive sensors by using a customized multimaterial 3D printer. The continuous conductive and dielectric layers achieve a substantial interfacial toughness of 1036 J·m^−2^. The sensors demonstrate a broad measurement range from 0.85 Pa to 5000 kPa. Fully 3D‐printed intelligent sensorized insole and soft robotic hand are demonstrated.

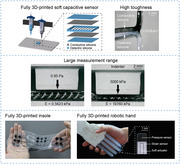

## 1 Introduction

A soft capacitive sensor typically consists of a soft dielectric layer sandwiched between compliant electrodes.^[^ ^1^, ^2^, ^3^, ^4^, ^5^, ^6^, ^7^, ^8^ ^]^ These sensors detect applied pressure or force by leveraging their deformable materials and structures. Due to its interesting attributes including good repeatability, low power consumption, high spatial resolution and low signal drift, soft capacitive sensors have extensive applications in wearable devices,^[^ ^1^, ^2^ ^]^ human‐machine interfaces,^[^ ^3^, ^4^ ^]^ flexible electronics,^[^ ^5^, ^6^ ^]^ soft robotics,^[^ ^7^, ^8^ ^]^ etc.

**Figure 1:**
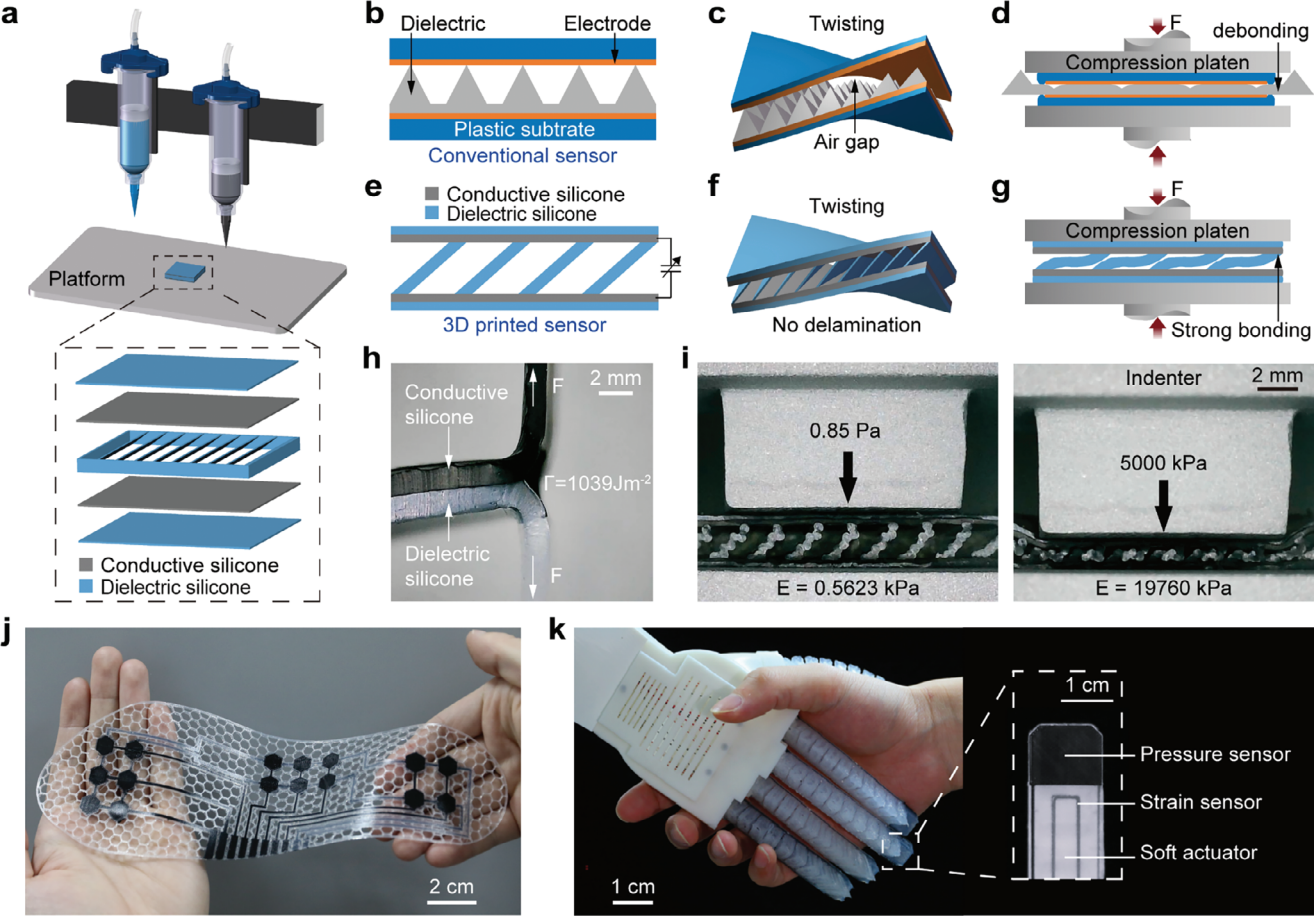
Fully 3D‐printed soft capacitive sensor of high toughness and large measurement range. a) Illustration of 3D‐printed soft capacitive sensor with tilted thin‐plates dielectric via DIW technique. b–d) Conventional soft capacitive sensors may suffer delamination and/or debonding. e–g) Fully 3D‐printed soft sensors have strongly bonded interfaces to resist both normal and shear stresses. h) The conductive silicone and dielectric silicone exhibit a high toughness of more than 1000 J·m^−2^ due to strong chemical crosslinks. i) The sensors can accurately measure a pressure within a large range from 0.85 Pa to 5000 kPa. j) Demonstration of fully 3D‐printed intelligent insole consisting of 16 soft sensors, which can perform real‐time monitoring of pressure distribution. k) Demonstration of fully 3D‐printed soft robotic hand combining soft pneumatic actuators, capacitive pressure sensors, and resistive strain sensors.

This paper focuses on fully 3D‐printed soft capacitive sensors of optimal design and performance (**Figure** **1**a). Traditionally, these sensors have been fabricated using methods such as mold casting, photolithography, screen printing, and sputtering^[^ ^1^, ^4^, ^9^, ^10^ ^]^ to achieve high sensitivity^[^ ^11^ ^]^ and thin profiles.^[^ ^1^ ^]^ However, manual assembly of cast segments could lead to durability issues such as delamination and debonding due to low interfacial toughness between layers.^[^ ^12^ ^]^ Additionally, microstructures like pyramids, domes, pillars, and porous designs have been integrated to enhance sensitivity by improving the compressibility of the dielectric material.^[^ ^13^, ^14^, ^15^ ^]^ However, when bending under a torque, these sensors might suffer delamination due to low stiction and small contact area between the dielectric and electrode layers (Figure 1b,c). Furthermore, using different materials for the dielectric and electrodes could result in a significant stiffness mismatch, leading to debonding under heavy compression loads (Figure 1d).^[^ ^11^, ^16^ ^]^

The delamination and debonding described above stem from the low interfacial toughness between the dielectric and electrode materials. Various chemical and material solutions have been proposed to address these issues, including swelling in a trichloromethane solvent,^[^ ^12^ ^]^ plasma treatment,^[^ ^17^ ^]^ high‐temperature imidization,^[^ ^18^ ^]^ dissolving PVA nanofibers^[^ ^1^ ^]^ and adding chromium (Cr) or SiO_2_ between layers.^[^ ^19^ ^]^ While these methods could enhance the interfacial toughness of the sensor up to 400 J·m^−2^,^[^ ^12^ ^]^ they involved complex procedures, manual assembly, and were time‐consuming.

The measurement range of a sensor is another significant property to demonstrate its capability and effectiveness. However, soft capacitive pressure sensors usually exhibit limited measurement range, due to the reasons as follows. First, as illustrated in Figure 1d, the sensor might suffer debonding at a large compression due to the stiffness mismatch, which limits its measurement range to be lower than 100 kPa.^[^ ^16^ ^]^ Second, the sensitivity of these sensors is heavily influenced by the stiffness of the dielectric layer.^[^ ^20^ ^]^ Sensors with low stiffness could detect small pressures (<1 Pa) effectively due to their deformability,^[^ ^7^, ^11^, ^12^, ^21^, ^22^ ^]^, but they might fail under high pressure or deformation, limiting their ability to measure pressures exceeding 1000 kPa. Conversely, sensors with higher stiffness could accurately measure high pressures (>1000 kPa),^[^ ^6^, ^23^, ^24^, ^25^, ^26^, ^27^ ^]^ but struggle with detecting small pressure variations (<1 Pa) due to their limited deformation capabilities. As we know, the dielectric material typically exhibits non‐linear behavior, and its stiffness varies at different strain levels. However, its variation range of stiffness is limited, which consequently limits the sensor's capability for a larger measurement range.

Advancing the field necessitates manufacturing techniques that integrate different materials with intricate architectures and strong adhesion.^[^ ^28^ ^]^ 3D printing techniques meet these demands by enabling the creation of highly complex 3D objects layer by layer. Fully 3D‐printed silicone‐based soft actuators and sensors have been demonstrated in previous studies.^[^ ^29^, ^30^, ^31^, ^32^, ^33^ ^]^ However, these works mainly focused on soft actuators^[^ ^29^, ^30^, ^31^ ^]^ and/or soft resistive sensors.^[^ ^32^, ^33^ ^]^ To the best of our knowledge, there is little work demonstrating fully 3D‐printed silicone‐based soft capacitive sensors. Previously, researchers have fabricated soft capacitive pressure sensors using a single material 3D printer, as a result, they could only print the dielectric layer^[^ ^34^, ^35^, ^36^, ^37^ ^]^ and electrode layers^[^ ^38^, ^39^, ^40^ ^]^ separately. These sensors generally had low interfacial toughness as they assembled the dielectric layer and electrode layers manually using 3M tape,^[^ ^34^, ^36^ ^]^ partially cured Ecoflex 00–30^[^ ^40^ ^]^ or simply stacked.^[^ ^35^, ^38^, ^39^ ^]^ In contrast, fully 3D‐printed soft capacitive sensors with microstructured dielectrics have been developed by using different materials (such as ionic elastomer, EPU, TPU, etc.) and different 3D technologies (such as DLP, FDM, etc.) to improve the sensitivity.^[^ ^41^, ^42^, ^43^, ^44^, ^45^ ^]^ Despite various materials being tested, fully 3D‐printed silicone‐based soft capacitive sensors have not been well studied. For example, a Digital Light Processing (DLP) 3D printer^[^ ^41^ ^]^ was used to print soft capacitive sensors and achieved high sensitivity. However, the dielectric is not stretchable, leading to mechanical property mismatches between the sensor materials, which can cause delamination under high pressure. Using a customized multimaterial DLP printer, soft capacitive sensors with multi‐mode sensing capabilities were printed.^[^ ^42^, ^43^ ^]^ However, issues such as ion leakage and water evaporation limited the detection of high pressure. Alternatively, TPU‐based soft capacitive sensors have been printed by multimaterial Direct Ink Writing (DIW)^[^ ^44^ ^]^ and Dual‐extruder Fused Deposition Modeling (FDM)^[^ ^45^ ^]^ 3D printers, but the high Young's modulus of TPU limits the detection range of the sensors, preventing them from detecting small pressures.

In this paper, we develop a customized multimaterial 3D printer to fabricate silicone‐based capacitive sensors with an optimal programmable design, capable of effectively resisting delamination and debonding under twisting and compression (Figure 1e–g). Our work is one of the first efforts to demonstrate fully 3D‐printed silicone‐based soft capacitive sensors. Our fully 3D‐printed silicone‐based soft capacitive sensors exhibit a high interfacial toughness (**Table** **1**) and a large measurement range (Figure 3j). The homogeneous silicone material system ensures similar stiffnesses between the electrode and dielectric layers, achieving an interfacial toughness of 1039 J·m^−2^ (Figure 1h). The introduction of separated tilted thin‐plate dielectrics reduces initial stiffness, thus enabling the sensor to detect small pressure. Therefore, the sensor demonstrates a wide measurement range from 0.85 Pa to 5000 kPa, enabled by the significant stiffness variation of the dielectric layer, ranging from 0.562 kPa to 19.76 Mpa (Figure 1i). We demonstrate a fully 3D‐printed intelligent insole that consists of 16 soft sensors, which can function effectively under 10000 cycles without any debonding and/or delamination (Figure 1j). We also 3D print a soft robotic hand that combines soft pneumatic actuators, capacitive pressure sensors, and resistive strain sensors with seamless integration (Figure 1k).

**Table 1:** Comparison in interfacial toughness between our 3D‐printed sensor with soft capacitive sensors in the literature.

Fabrication method	Interfacial toughness [J·m^−2^]		References
	Flat interface	Microstructure interface	
Molding	420	390	[12]
Molding	370	386	[17]
3D printing	339.3	N/A	[42]
3D printing	1000	N/A	[43]
3D printing	75	N/A	[37]
3D printing	1036	645 (Transverse), 339 (Longitudinal)	This work

# N/A, Not available.

## 2 Results

### 2.1 Fully 3D‐Printed Soft Capacitive Sensor of High Toughness and Large Measurement Range

A customized direct ink writing (DIW) 3D printer (Figure S1, Supporting Information) is developed to fabricate soft capacitive sensors of optimal material and structural designs (Figure 1a). By adjusting the compositions of dielectric and conductive inks, we ensure that both the conductive and dielectric silicones in the sensors have similar stiffness properties, thereby minimizing material property mismatches.

Different from conventional soft capacitive sensors which may suffer delamination and/or debonding (Figure 1b–d), our 3D‐printed soft sensor mainly consists of separated tilted thin‐plate dielectrics silicones as the intermediate elastomer, sandwiched between two planar conductive silicones (Figure 1d). We compare five designs, and finally choose an optimal one (Figure S2, Supporting Information). The comparison among five structure designs is shown in Table S1 (Supporting Information).

During the 3D printing process, both the conductive and dielectric silicone inks remain uncured, facilitating the formation of strong molecular networks and chemical bonds at the interfaces between the electrodes and dielectric. Consequently, the sensor can resist delamination under the deformation of twisting (Figure 1f). Additionally, when subjected to normal forces or pressure, both the electrodes and dielectric undergo similar deformations due to their closely matched stiffness properties, enhancing the sensor's resistance to debonding (Figure 1g). The Young's moduli of conductive and dielectric silicones are found to be 840 kPa and 580 kPa, respectively, and the interfacial toughness of the printed conductive and dielectric silicones is 1039 J·m^−2^ (Figure 1h). The sensor also exhibits a large measurement range from 0.85 Pa to 5,000 kPa (Figure 1i). All the properties will be discussed later.

We demonstrate a fully 3D‐printed intelligent insole that consists of 16 soft sensors, which can function effectively under 10000 cycles without any debonding and/or delamination (Figure 1j). Furthermore, we showcase a 3D‐printed soft robotic hand that combines soft pneumatic actuators, capacitive pressure sensors, and resistive strain sensors with seamless integration (Figure 1k).

### 2.2 Ink Design for Direct Ink Writing

Both dielectric and conductive silicones are explored to develop fully printable silicone‐based capacitive sensors, with a particular focus on optimizing the microstructured dielectric layer. These inks are engineered with specific viscoelastic properties tailored for effective 3D printing. During printing, they exhibit shear‐thinning behavior, ensuring smooth flow through the nozzle with shear stress (τ) exceeding the yield stress (τ_y_). Upon exiting the nozzle, they transition to a solid‐like state with a storage modulus (G′) higher than the loss modulus (G″) crucial for maintaining structural integrity according to the desired mechanical design principles.^[^ ^46^, ^47^ ^]^ Both the dielectric and conductive inks have sufficiently high yield strength, tens of Pascals or higher,^[^ ^29^, ^48^ ^]^ enabling them to support the printing of overhangs.

**Figure 2:**
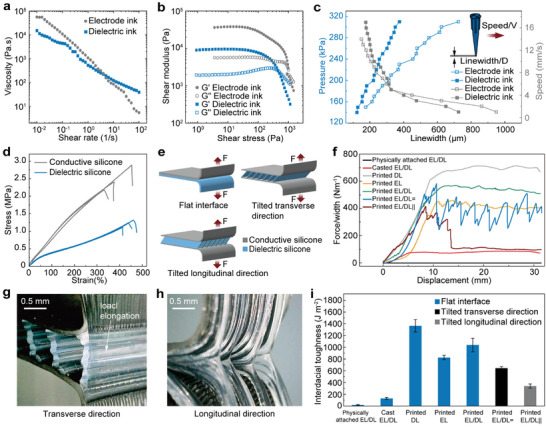
Rheological properties of printable inks and interfacial properties of fully 3D‐printed soft sensors. a) Apparent viscosity of the electrode or dielectric ink as a function of shear rate. b) Storage and loss moduli (G' and G'') of the electrode or dielectric ink as a function of shear stress. c) Applied pressure and printing speed as a function of the linewidth of the printed electrode or dielectric ink. The nozzle has an inner diameter of 210 um. d) Tensile stress‐strain curve of the printed conductive or dielectric silicone. e) Schematic of three peel tests. The first test is on a printed sensor with a solid dielectric. The second and third tests are on a printed sensor with multiple separated tilted thin‐plates dielectrics in the transverse and longitudinal directions, respectively. f) Peel force as a function of displacement for conductive and dielectric silicones with different configurations and/or based on different fabrication methods. g,h) Snapshots of the peel tests on the 3D‐printed soft sensor with multiple separated tilted thin‐plates dielectrics in the transverse and longitudinal directions, respectively. i) Interfacial toughness of conductive and dielectric silicones with different configurations and/or based on different fabrication methods.

Previous studies have utilized Ecoflex‐0030 as dielectric silicones due to their low stiffness.^[^ ^40^ ^]^ However, these materials are prone to collapse, difficult to print the tilted thin‐plate structures. While PDMS^[^ ^36^ ^]^ (SE1700) facilitates microstructure printing, its high stiffness restricts sensor sensitivity. Single‐component moisture‐curing silicones have been used to print self‐support microfluidic devices,^[^ ^49^ ^]^ but it has not yet been explored in 3D printing silicone‐based soft capacitive sensors with microstructured dielectrics. We select LOCTITE SI 595 CL because this ink demonstrates shear‐thinning and shear‐yielding behavior, crucial for effective extrusion and printing (**Figure** **2**a). Its high plateaus of storage modulus (G′) and shear yield stress (τ_y_) in the uncured state, as indicated in Figure 2b, are important for maintaining the predetermined shape and minimizing the structural distortion. For example, the maximum G′ of 9.1 kPa suggests that the silicone ink has good elasticity to maintain its printed shape. Notably, the increase in τ_y_ (0.45 kPa) during curing when exposed to air suggests the ink's ability to withstand self‐gravity and maintain structural integrity over time.

For the electrode ink, mixing PDMS with a large amount of carbon nanotubes (12 wt.%) can improve the shear yield stress(0.4 kPa) and exhibit shear‐thinning behavior.^[^ ^40^ ^]^ However, the shear yield stress is still not sufficient to print overhang^[^ ^40^ ^]^ and further increases in carbon nanotube content can lead to nozzle clogging.

We use ELASTOSIL LR 3162 (Wacker), a paste‐like, two‐component electrically conductive liquid silicone that has not been used in 3D printing before. It has a dynamic viscosity of 5,700 Pa·s at the shear rate of 1 s^−1^, which is usually too high for the DIW method and may suffer nozzle clogging. To enhance its printability, Silicone Thinner WS‐DA‐G20 (Shanghai Wellion) is added into Part A and Part B of ELASTOSIL LR 3162 separately with a weight ratio of 28.6 wt.% to decrease the viscosity of the electrode ink. The electrode ink also exhibits shear‐shinning and shear‐yielding (Figure 2a). Compared to the dielectric ink, the electrode ink has even higher plateaus of G′ (37.8 kPa) and τ_y_ (0.98 kPa), allowing for 3D printing overhang structures (Figure 2b).

Taking advantage of the inks with the above material properties, we can print soft capacitive sensors with self‐supporting tilted thin plates in a single step without supporting materials. The pot life of this electrode ink is excellent, that is, even after being mixed and stored at room temperature for more than 24 h, its rheological properties do not significantly change, although the shear yield stress may increase slightly (Figure S3, Supporting Information). This observation ensures long‐time 3D printing of large‐volume structures.

In order to achieve stable 3D printing with continuous and uniform filaments of the dielectric and electrode inks, we conduct a printability analysis to optimize the printing parameters focusing on the nozzle movement speed and the applied pressure on the inks. The diameter of the printed fiber is measured by a digital microscope system (Leica DMS300). As shown in Figure 2c, we calibrate the linewidth (D) of the dielectric or conductive silicone ink, where the nozzle's diameter is 210 um and the layer height is kept constant at 0.2 mm. As we can see, at a nozzle planar movement speed of 10 mm s^−1^, for the dielectric silicone ink, the linewidth increases from 0.1225 mm to 0.375 mm as the extrusion pressure increases from 140 to 310 kPa. On the other hand, with a constant extrusion pressure of 220 kPa, for the dielectric silicone ink, the linewidth decreases from 0.725 to 0.175 mm as the nozzle movement speed increases from 1 to 14 mm s^−1^. The conductive silicone ink performs a similar behavior to the dielectric one. These results indicate that the line width can be tuned by varying the nozzle movement speeds and extrusion pressures, allowing careful control to print tilted thin plates in a single step without supporting materials.

Figure 2d shows the stress‐strain curve of the conductive or dielectric silicone. The Young's moduli of conductive or dielectric silicone are 840 and 580 kPa, respectively. Both conductive or dielectric silicone rubber have more than 350% elongation at break. The equivalent interfacial toughness with intermediate microstructure can be improved by increasing the elongation at break. The initial conductivity of the cured electrode ink is 5.91 S·m^−1^ and remains above 1.07 S·m^−1^ within a strain range of up to 200% (Figure S4, Supporting Information). The decrease in conductivity might affect the sensor's response time, but this could be improved by using a kirigami‐inspired electrode design^[^ ^50^ ^]^ or different electrode materials^[^ ^51^ ^]^ to achieve strain insensitivity.

### 2.3 Interfacial Properties of Fully 3D‐Printed Soft Sensors

We conduct a detailed analysis of the interfacial properties of fully 3D‐printed soft sensors, focusing on three types of peel tests. These tests examine the interfaces between solid conductive silicone and solid dielectric silicone, and between conductive silicone and tilted thin‐plates dielectric silicone in both transverse and longitudinal orientations (Figure 2e).

The experimental peel force as a function of the displacement is shown in Figure 2f when two electrode layers (EL) are 3D‐printed continuously, the interfacial toughness between the EL is 825 J·m^−2^. In addition, when two dielectric layers (DL) are 3D‐printed continuously, the interfacial toughness between two flat DLs is higher, that is, 1365 J·m^−2^. This indicates that the DL has a stronger interfacial bonding strength compared to the EL.

Furthermore, the interfacial toughness between the flat EL and DL, when the two layers are 3D‐printed together, is 1039 J·m^−2^. In contrast, using a casting method to create separate EL and DL layers, and then bonding them with a PDMS adhesive (in a 10:1 weight ratio), resulted in a significantly lower interfacial toughness of 129 J·m^−2^ after curing. This disparity underscores the superior interfacial bonding achieved through 3D printing, where uncured conductive and dielectric silicone inks form robust molecular networks and chemical bonds post‐printing, resulting in higher toughness at the electrode‐dielectric interfaces.

In addition, it is worth noting that when the EL and DL are physically attached without any adhesive, the interfacial toughness is zero. This multilayer stacking method^[^ ^16^, ^52^, ^53^, ^54^ ^]^ is widely used in the development of soft capacitive sensors, the functional layers are stacked on top of each other without bonding, since it may generate a subtly changed interface, leading to high sensitivity. However, the structures, developed by this method, can be unstable.

To enhance the sensitivity of the 3D‐printed sensor, we implement multiple separated tilted thin‐plate dielectrics as the intermediate layer. Due to its anisotropic structure as shown in Figure 2g,h, the sensor exhibits different interfacial toughnesses in the transverse and horizontal directions, respectively. For example, the interfacial toughness is 645 J·m^−2^ or 339 J·m^−2^ in the transverse or longitudinal direction, respectively (Figure 2f,i). As we can see, in the transverse direction, the peel force exhibits a zigzag pattern, that is, the force increases when the crack is pinned, but may decrease until another new crack is initiated (Figure S5, Supporting Information). This observation is attributed to the presence of the tilted thin plates in the intermediate dielectric layer. These tilted thin plates can elongate out of the plane to dissipate the strain energy, functioning as multiple elastic dissipaters. Consequently, larger elongation at fracture results in higher interfacial toughness. The high interfacial toughness is consistent with the excellent stretchability (with strain over 350%) of both the conductive and dielectric silicones, as shown in Figure 2d. This elastic energy dissipation can also be verified by optical microscopic images. As shown in Figure 2g, large local elongation of the middle layer can lead to substantial energy dissipation. In the longitudinal direction, the peel force initially increases until a crack is formed, and then the crack may propagate rapidly (Figure 2h). In general, 3D printing of both the EL and DL together in a single process can result in a stronger interfacial toughness compared to the traditional casting method. This enhanced interface bonding leads to the overall performance and durability of the soft capacitive sensors.

We compare the interfacial toughness with previous soft capacitive sensors,^[^ ^12^, ^17^, ^37^, ^42^, ^43^ ^]^ as shown in Table 1. The highest interfacial toughness between the dielectric and conductive layers could reach up to 1000 J·m^−2^,^[^ ^43^ ^]^ but their sensor's detection range is limited to 5 Pa to 550 kPa. In comparison, our work achieves a detection range of 0.85 Pa to 5 MPa. The interfacial toughness between dielectric and conductive layers is 1039 J·m^−2^, and the sensor exhibits interfacial toughness values of 645 J·m^−2^ (transverse) or 339 J·m^−2^ (longitudinal), which are comparable to those of highly durable non‐3D‐printed capacitive sensors (390 J·m^−2^).^[^ ^12^ ^]^

### 2.4 Large Measurement Range of Fully 3D‐Printed Soft Sensor

Various microstructures for the dielectric layer are tested, including tilted thin‐plate, stack, honeycomb, solid, and pyramid configurations, to explore the optimal design of the fully printable capacitive sensor (Figure S2, Supporting Information). The sensor with tilted thin‐plate dielectrics demonstrates the best performance in terms of sensitivity, measurement range, and interfacial toughness (Table S1, Supporting Information).

**Figure 3:**
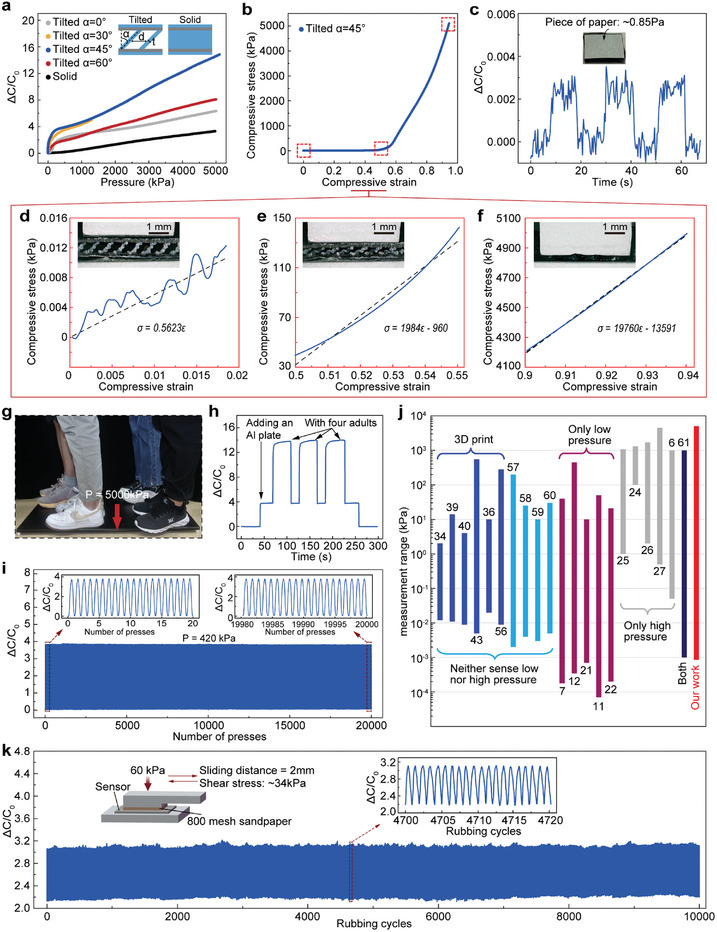
Large measurement range of fully 3D‐printed soft sensor. a) Capacitance change as a function of applied pressure for the sensors with five programmable dielectrics. The sensor with tilted 45° dielectric layers exhibits the best sensing performance. b) Compressive stress‐strain curve of the sensor with tilted 45° dielectric layers. c) Detection limit of the sensor. The sensor can sense a piece of paper with a small pressure of 0.85 Pa (inset). d–f) Zoomed stress‐strain curve in the small, middle, or large pressure interval, respectively. The inset shows a snapshot of the sensor's cross section. g) The sensor is under a large load of 5000 kPa, which consists of an aluminum plate and four adults. h) Capacitance change of the sensor under a large load of 5000 kPa. i) Cycling stability at a pressure of 420 kPa (over 20000 cycles). The insets show the first 20 cycles and the last 20 cycles. j) Comparison of soft capacitive sensors in the literature, in the aspects of measurement range. k) Cyclic rubbing test (10,000 cycles) combining a normal pressure (60kPa) and a shear stress (34kPa). The inset shows a schematic of the experimental setup.

**Figure** **3**a shows the capacitance change as a function of the applied pressure for the soft sensors with multiple separated tilted thin‐plate dielectrics. For each sensor, we keep its area as 10 mm × 10 mm and thickness as 1.8 mm, but vary the following structural parameters including the tilted angle (*α*), horizontal spacing (*d*), and thickness of the thin wall (*t*), as illustrated in the subfigure in Figure 3a. The sensor with a solid elastomer is also considered for comparison. It is found that all the sensors with separated tilted thin‐plate dielectrics exhibit higher sensitivity, compared to that with the solid elastomer, where the sensitivity of a capacitive sensor is defined as S = δ(ΔC/C_0_)/δP, C_0_ is the initial capacitance without any loading of pressure, and ΔC is the capacitance change when a pressure P is applied. In particular, the sensor with *α* = 45°, *d* = 1.5 mm, and *t* = 0.2 mm exhibits the highest sensitivity. It should be noted that we do not use any supporting materials during 3D printing, in order to simplify the printing process and decrease both the material and time costs. Accordingly, the structural parameters should be carefully designed and optimized to prevent the top electrode and/or the tilted thin plates from collapsing. In general, increasing the tilted angle requires thicker thin plates to resist gravity effects. As shown in Figure 3a, with *α* = 45°, *d* = 1.5 mm, *t* = 0.2 mm, the sensor's sensitivity is 0.22 kPa^−1^ when 0 < *p* < 5 kPa, 0.01 kPa^−1^ when 5 kPa < *p* < 300 kPa, 0.0025 kPa^−1^ when 300 kPa < *p* < 5000 kPa, respectively. In comparison, for the sensor with a solid dielectric, its sensitivity is 0.0007 kPa^−1^ when 0 < *p* < 5000 kPa. When the applied pressure is small, the sensitivity of a soft sensor with multiple separated thin‐plate dielectrics is 314 times higher than that of a soft sensor with a solid dielectric layer. This observation can be interpreted as follows. A soft capacitive sensor with large air space in the middle elastomer can significantly increase its compliance, so that the sensor is easy to deform when subject to a compressive load and thus has a high sensitivity.

As shown in Figure 3a, the sensor reaches its highest sensitivity at a tilted angle of α = 45°. While increasing α enhances sensitivity, it also requires widening the extrusion to prevent collapse during printing. Although sensitivity improves as α increases from 0° to 30° to 45°, further increases in α require thicker tilted thin plates, which reduce the air space in the dielectric layer and lower sensitivity.

It is worth noting that with *α* = 30°, *d* = 1.5 mm, *t* = 0.2 mm, the detection range of the sensor is only 1300 kPa, which is due to a short circuit at high pressure.

Figure 3b shows the experimental compressive stress‐strain curve of the soft sensor with optimal design (*α* = 45°, *d* = 1.5 mm, *t* = 0.2 mm). The sensor exhibits variable stiffness at different strain ranges. At a small strain of less than 2%, the sensor can sense a piece of paper with a small pressure of 0.85 Pa (Figure 3c; Figure S6; Movie S1, Supporting Information). To avoid any interference from hand proximity, we used a wooden stick for the pick‐and‐place procedure.

In contrast, a sensor with a solid dielectric has a detection limit of 200 Pa (Figure S7, Supporting Information). The reason is that the sensor with thin‐plate dielectrics has a small compressive modulus of 0.5623 kPa due to the large air space in the middle thin‐plate elastomer (Figure 3d). At an intermediate strain within 50% to 55%, the sensor's modulus increases to 1.984 MPa, with air space still available in the elastomer (Figure 3e). At a large strain within 90% to 94%, the thin‐plates dielectrics are compressed into a nearly solid elastomer with negligible air, resulting in a modulus of 19.76 MPa, 10 times that at an intermediate strain range, and 35141 times that at a small strain range (Figure 3f).

It should be emphasized that the sensitivity may strongly depend on the sensor's stiffness. For a soft capacitive sensor with a solid dielectric, assuming the elastomer behaves according to a linear and incompressible model, sensitivity can be inversely proportional to its compressive stiffness, see the detailed derivation in Methods. That is to say, in order to achieve a small detection limit, that is, a high sensitivity at the small strain range, the material should have a very small modulus. However, if the material is too soft, the material cannot sustain a large pressure and/or deformation. That is to say, in order to achieve a large measurement range, the material can have a small modulus at the small strain range but a large modulus at the large strain range. Taking advantage of this tilted thin‐plate structure, the elastomer has a compressive modulus of 0.5623 kPa at the small strain range (Figure 3d), which is softer compared to most soft materials in the literature.^[^ ^55^ ^]^ However, when the load is high, its stiffness can increase by 35141 times to sustain a large strain. Consequently, this soft sensor has a large measurement range from 0.85 Pa – 5000 kPa. The changes in capacitance and strain of our sensor are shown in Movie S2 (Supporting Information) when the sensor is loaded within a large pressure range until 5000 kPa.

To further validate the sensor's capability for measuring high pressure, we conduct experiments as illustrated in Figure 3g, where the sensor is placed under an aluminum plate supported by five dots each with an area of 1cm^2^. The contact area between the aluminum plate and the ground is 5 cm^2^ (Figure S8, Supporting Information). Four adults, with a total weight of ≈250 kg, stand on the aluminum plate, resulting in a pressure of 5000 kPa on the sensor. The capacitance change of the sensor is illustrated in Figure 3h. At high pressure, the increase in capacitance of the sensor is not only due to the reduced separation between the electrodes but the enlarged electrode area as well, as shown in Movie S2 (Supporting Information).

Our 3D‐printed capacitive sensor is also capable of detecting intermediate pressures, as shown in Figure S9 (Supporting Information). Under five cycles of periodic pressure, with an amplitude of 50, 2100, or 4300 kPa, the sensor always exhibits a consistent stable response. In addition, the time scale for creep and relaxation is an important sensing parameter related to the viscoelastic property of the soft sensor. In the experiments, we apply, hold, and then remove a pressure of 5 kPa, and the creep and relaxation time is found to be 6.6 ms (Figure S10, Supporting Information). Furthermore, the pressure resolution is another critical parameter to determine the sensor's ability to detect a small variation in pressure after an initial pressure P_0_ is applied. When P_0_ is set to be 5, 800, or 4000 kPa, the sensor demonstrates a resolution of 60 Pa (1.2%), 18 kPa (2.25%), or 150 kPa (3.75%), respectively (Figure S11, Supporting Information). To further validate the pressure resolution, we also test the pressure sensor of area 1cm^2^ by placing it under a car tire. With a mass of 2000 kg, the car generates an initial pressure of ≈280 kPa on the sensor. Remarkably, the sensor can detect the change of pressure (i.e., 8.3 kPa) when a passenger of 60 kg boards or leaves the vehicle (Figure S12, Supporting Information). The sensor also exhibits exceptional stability during cyclic testing. Under a pressure variation of 0 – 420 kPa for over 20,000 cycles, the sensor remains remarkably stable without any significant change in signal output, as shown in Figure 3i. In more extreme conditions (Figure 3k), our 3D‐printed sensors sustain over 10,000 cycles in rubbing tests under simultaneous normal force (60 kPa) and shear force (34 kPa).

Figure 3j and Table S2 (Supporting Information) compare the measurement range of our 3D‐printed sensor with those of other soft capacitive sensors in the literature. Specifically, compared to other 3D‐printed soft capacitive pressure sensors^[^ ^34^, ^36^, ^39^, ^40^, ^43^, ^56^ ^]^ and commonly used soft capacitive sensors,^[^ ^57^, ^58^, ^59^, ^60^ ^]^ our sensor has the smallest detection limit (0.85 Pa) and the largest measurement pressure (5000 kPa). Some soft capacitive sensors have a small detection limit (<1 Pa) but are unable to detect high pressure (<1000 kPa).^[^ ^7^, ^11^, ^12^, ^21^, ^22^ ^]^ Conversely, others can detect high pressure (>1000 kPa) but do lack sensitivity in the low pressure range (<1 Pa).^[^ ^6^, ^24^, ^25^, ^26^, ^27^ ^]^ Only a limited number of soft capacitive sensors can cover both pressure ranges (<1 Pa and >1000 kPa),^[^ ^61^ ^]^ but these sensors typically have a narrower overall detection range compared to ours. We also compare the measurement range of our 3D‐printed sensor with iontronic pressure sensors (Table S3, Supporting Information). Our sensors still have the widest detection range.

In summary, because the conductive silicone and dielectric silicone used have similar Young's modulus and the same Poisson's ratio, the sensor does not suffer from mechanical mismatch issues. Additionally, the sensor's compressive modulus changes by a factor of 35141 (from 0.5623 kPa to 19.76 MPa) under pressures ranging from 0 to 5000 kPa. These two factors contribute to the sensor's large detection range.

### 2.5 Fully 3D‐Printed Personalized Insole with Extraordinary Durability

3D‐printed soft sensors can be integrated with wearable devices to improve their functionalities. In this work, we demonstrate a specific application of a fully 3D‐printed personalized insole that can perform real‐time pressure monitoring with extraordinary durability for 10000 cycles. Previous studies showed that soft sensors, embedded in insoles, could monitor static and dynamic plantar pressure mapping. However, they were usually hand‐made, involved complicated processes, and were difficult to achieve customized demands.^[^ ^62^, ^63^, ^64^ ^]^ Efforts toward 3D‐printed insoles with embedded pressure sensors have been made, yet these often employed a limited number of sensors (≤8), restricting their ability to accurately map pressure.^[^ ^65^, ^66^ ^]^ Additionally, some printed sensor exhibited low sensitivity (<0.0001 kPa^−1^).^[^ ^44^ ^]^ In particular, the durability of insoles, which usually involves delamination and/or debonding of the sensors, is not carefully examined especially when the insole is under complicated loading conditions with both normal and shear stresses.

**Figure 4:**
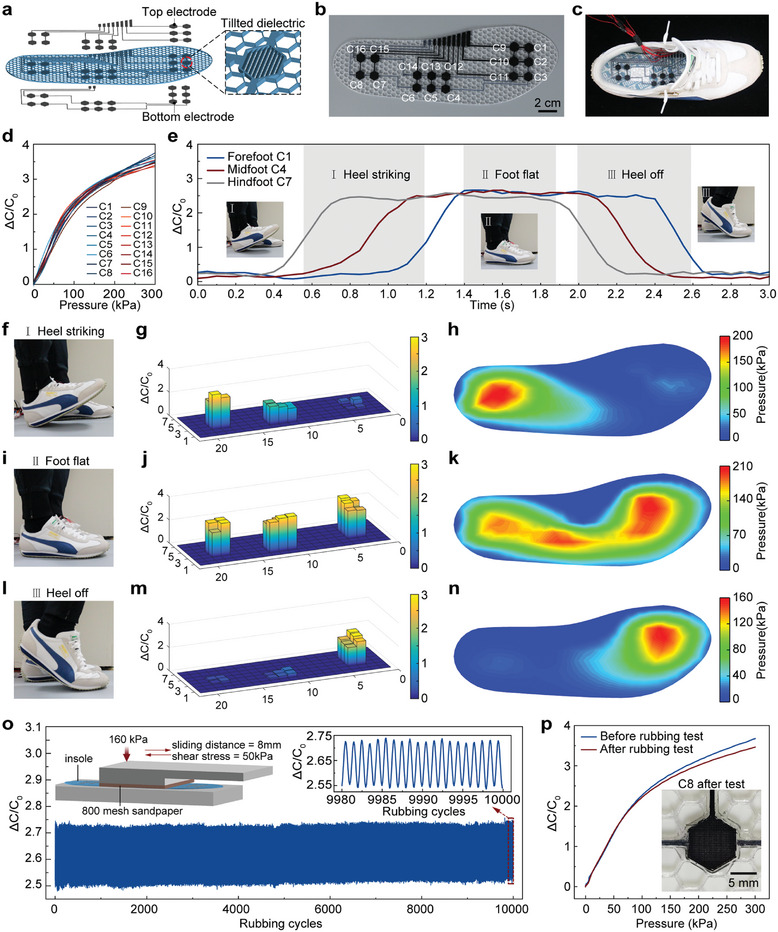
Fully 3D‐printed insole consisting of an array of 16 soft pressure sensors. a) Design of the insole and distribution of the sensors. b) Image of the fully 3D‐printed insole. c) The insole is placed into a shoe for real‐time pressure monitoring. d) Capacitance change as a function of applied pressure of the 16 sensors. The characterization curves of these sensors are close to each other. e) The 3D‐printed intelligent insole can perform real‐time pressure monitoring and then conduct gait analysis. f–h) Figure 4f–h) The foot state, sensor reading, and pressure distribution when the heel strikes the ground. The pressure distribution is obtained through interpolation of sensor data. i–k) The foot state, sensor reading, and pressure distribution when the foot is flat. l–n) The foot state, sensor reading, and pressure distribution when the heel leaves the ground. o) Cyclic rubbing test (over 10,000 cycles) combining a normal pressure (160 kPa) and a shear stress (50 kPa). The inset shows a schematic of the experimental setup. p) Capacitance change as a function of applied pressure of the sensor C8 before and after over 10,000 cycles rubbing test.

We print an insole of length 24 cm and width 8.5 cm, together with an array of 16 soft capacitive sensors. As shown in **Figure** **4**a, each sensor consists of a bottom electrode, multiple separated tilted thin‐plate dielectrics, and a top electrode. In this array, 6 sensors are placed on the forefoot, 6 sensors on the midfoot, and 4 sensors on the heel (Figure 4b). After the insole is put into the shoe (Figure 4c), we measure the capacitance of each sensor in the array, by using a capacitance to digital converter (CDC) and two multiplexers. We calibrate these 16 soft sensors in the insole, and their performance is found to be close and consistent, as shown in Figure 4d. In addition, their sensitivity (≈0.03 kPa^−1^ at 50 kPa) is much larger than the soft sensors in previous studies,^[^ ^44^, ^66^ ^]^ due to their optimal design and low modulus, ensuring accurately real‐time monitoring of pressure distribution in the insole.

We then conduct dynamic experiments on this sensorized insole. During this test, the heel strikes the ground first, followed by the contact of the whole foot, and finally the forefoot strikes the ground. Figure 4e shows the pressure variations of three typical sensors (i.e., C1, C4 and C7) in the heel, midfoot, and forefoot regions, respectively. The foot motion and the data from the sensors are also demonstrated in Movie S3 (Supporting Information). As we can see, this intelligent insole can effectively perform real‐time pressure monitoring, and the readings from the sensors can be used for the foot posture analysis, as illustrated in Figure 4f–n. For example, at the moment when the heel strikes the ground (Figure 4f), the readings from all 16 sensors can be recorded in a real‐time manner (Figure 4g). Based on these data, a heatmap can be generated to provide a visual representation of the pressure distribution on the plantar surface corresponding to any specific foot posture (Figure 4h). Studying foot pressure helps understand biomechanics, pinpoint abnormal pressure areas, and design interventions for foot problems.^[^ ^67^ ^]^ It can also offer valuable assistance to individuals with diabetes by continuously monitoring and alerting them to excessive pressure or potential ulceration areas on their feet.^[^ ^68^ ^]^

The sensor array in the insoles experiences crosstalk, and the multiplexing module introduces noise, resulting in an increased signal‐to‐noise ratio during testing. By employing a high‐precision multiplexing module,^[^ ^8^ ^]^ noise can be reduced. Additionally, designing a circuit with a compensation algorithm^[^ ^17^ ^]^ can effectively address crosstalk in sensor arrays.

Furthermore, the 3D‐printed personalized insole demonstrates consistent signal output even under more complicated and extreme testing conditions. For instance, during cyclic rubbing testing, where an indenter moves back and forth over the sensor at a speed of 2 mm s^−1^ with an 8 mm displacement, the sensor withstands both a normal pressure of 160 kPa and a shear stress of 50 kPa (Figure 4o). Remarkably, the sensor maintains a stable signal output throughout the rubbing test over 10,000 cycles, and the capacitance change as a function of the applied pressure, for the sensor C8, has little change after the rubbing test (Figure 4p). This sensorized insole exhibits extraordinary durability and stability, attributing to the large interfacial toughness between our 3D‐printed dielectric and conductive silicones even under complicated and extreme loading conditions.

### 2.6 Fully 3D‐Printed Robotic Hand Combining Soft Actuators and Soft Sensors

**Figure 5:**
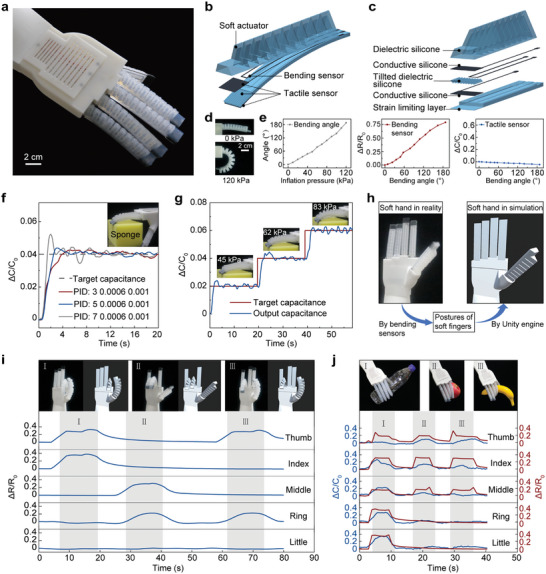
Fully 3D‐printed soft robotic hand combining soft actuators and soft sensors. a) Image of soft robotic hand. b) Each soft finger consists of soft pneumatic actuators, capacitive tactile sensors and resistive bending sensors. c) Detailed structure of soft finger. d) Images of soft finger at 0 kPa (top) and 120 kPa (bottom). e) Response of the bending angle, bending sensor and tactile sensor when the inflation pressure is applied to the pneumatic actuators in the soft finger. f) PID force control when the soft finger touches a sponge. The inset shows the tuning parameters. g) Multilevel tactile feedback during continuous pressing on a sponge. h) Experimental setup for controlling virtual robotic hand using bending sensors in soft robotic hand. i) Real time gesture recognition of soft robotic hand using bending sensors. j) Grabbing objects of different shapes using both the tactile and bending sensors.

We then develop a fully 3D‐printed soft robotic hand combining soft actuators and soft sensors, as shown in **Figure** **5**a. The soft robotic hand is composed of five fingers and a palm. Each finger consists of soft pneumatic actuators, capacitive tactile sensors, and resistive bending sensors (Figure 5b), and is made of dielectric and conductive silicones (Figure 5c). These soft fingers are fabricated by using our multimaterial 3D printer in one step, eliminating the needs for post‐processing.

To fabricate these soft fingers, we adjust printing parameters during the slicing process. This involves increasing the line width, reducing the layer height, and utilizing a continuous Eulerian tool path^[^ ^69^ ^]^ (Figure S13, Supporting Information). See Table S4 (Supporting Information) for the details about parameter setting. These optimizations notably improve the consistency of the fingers in terms of both material and structure (Figure S14a, Supporting Information). The bending sensors at the bottom of each finger are clearly visible (Figure S14b, Supporting Information). Our current fingers exhibit smoother surfaces compared to those without optimized printing paths (Figure S15, Supporting Information). Importantly, there is no interference between the conductive and dielectric silicones, ensuring effective functionality of both the soft actuators and sensors.

As illustrated in Figure 5b, the tactile sensor is placed at the tip of the soft finger, and the bending sensor is along the length of the finger. The tactile sensor is essentially a soft capacitive sensor consisting of multiple tilted dielectrics as discussed above, while the bending sensor is a soft resistor made of conductive silicone (Figure 5c). The five tactile sensors can detect applied forces and decoupling them from each other (Movie S4; Figure S16, Supporting Information). Within a large strain range of 0–300%, the resistive sensor maintains a consistent gauge factor (GF) of 5.7, where the GF is defined as ΔR/(R_0_·e), ΔR is the resistance change when a loading is applied, R_0_ is the initial resistance without any loading, and e is the engineering strain (Figure S17, Supporting Information). This resistive sensor exhibits excellent repeatability over 10,000 cycles, as illustrated in Figure S18 (Supporting Information).

Each soft finger bends when the air chamber in the soft actuator is loaded with an air pressure (Figure 5d). As the pressure increases to 120 kPa, the finger's bending angle turns to 188°, resulting in a gradual increase in the resistance of the bending sensor, while the capacitance of the tactile sensors remains nearly unchanged (Figure 5e). Since the tactile sensor is placed at the finger's tip, it is actually decoupled with the bending sensor. Consequently, the bending sensor mainly detects the bending of the soft finger, while the tactile sensor measures the contact forces applied on the fingertip.

With the tactile sensors in the fingertips, the soft robotic hand can achieve real‐time closed‐loop feedback control of the output force when grasping an object. A conventional proportional‐integral‐derivative (PID) controller (the structure is shown in Figure S19, Supporting Information) is then designed when the finger touches a soft sponge (Figure 5f), and multilevel tactile feedback control can also be accurately performed (Figure 5g). With these soft tactile sensors, the soft robotic hand can further achieve force‐controlled grasping of fragile or highly deformable objects such as a cupcake case (Figure S20; Movie S5, Supporting Information). Without these sensors, it is challenging for the fingers to regulate the inflation pressures to ensure secure grasping and meanwhile avoid large deformation (which may lead to structural instability) of the cupcake case (Figure S20b, Supporting Information).

Taking advantage of the soft bending sensors, this soft robotic hand can be mimicked by a simulated robotic hand in virtual reality. As illustrated in Figure 5h, the soft bending sensors can monitor the posture of the soft hand. According to these data, a virtual robotic hand is then constructed in Unity3D.^[^ ^70^ ^]^ In simulating this robotic hand, we divide each finger into multiple segments and assume that each segment has the same bending curvature (Figure S21, Supporting Information). Consequently, the virtual hand can repeat the posture of our soft robotic hand in a real‐time manner (Figure 5I; Movie S6, Supporting Information). The accurate simulation in virtual reality may have extensive applications in medical training,^[^ ^71^ ^]^ metaverse^[^ ^72^ ^]^ and human‐machine interaction.^[^ ^73^ ^]^

Lastly, we demonstrate that the soft robotic hand can grab objects of different shapes effectively, based on the fusion of these tactile and bending sensors. In grasping a cylindrical water bottle (200 g), as well as simulated models of a banana (53 g) and an apple (21 g), the soft robotic hand can automatically discriminate the object's shape through capacitive and resistive changes (Figure 5j; Movie S7, Supporting Information). The fusion of sensors can empower a soft robot or device with capability to perform effective operations in unstructured environments.^[^ ^74^ ^]^

Currently, due to the limited output force of the pneumatic actuator, it is unable to grasp heavier objects. In future work, we aim to enhance the grasping force of the soft robotic hand.

## 3 Conclusion

In summary, we develop a customized multimaterial 3D printer to fabricate soft silicone‐based capacitive sensors in a single printing process. The fully printable soft sensors demonstrate a high interfacial toughness (i.e., 1036 J·m^−2^) between the conductive and dielectric silicone layers, with both materials exhibiting similar Young's modulus and significant stretchability. In particular, the soft sensor of optimal design, with multiple tilted thin‐plate dielectrics as the intermediate elastomer, exhibits a large measurement range (i.e., from 0.85 Pa to 5000 kPa), due to its extreme stiffness variation range (i.e., the ratio of the maximum to minimum stiffnesses is 35341). We demonstrate two applications of this fully printable soft sensor. The first is an intelligent personalized insole embedded with a sensor array, which can be 3D‐printed in one step. This sensorized insole allows real‐time monitoring of plantar pressure distribution, facilitating gait analysis, and shows exceptional durability, enduring over 10,000 cycles of complex loading conditions involving both normal and shear stresses without delamination and/or debonding failure. The second application is a 3D‐printed robotic hand combining both soft actuators and soft sensors. With association of sensor fusion, the robotic hand can perform various tasks, such as conducting feedback control, recognizing its posture for simulation/analysis, and grasping objects of various shapes.

We believe that our strategy of employing 3D printing for soft microstructured sensors represents a general approach that not only enhances the performance of soft sensors but also facilitates the design of robust soft functional devices. The seamless integration of soft sensors and soft actuators can significantly enhance the functionalities of soft robotic systems, enabling them to perform diverse tasks with precise sensing and control, particularly in challenging, unstructured environments.

## 4 Experimental Section

### Dielectric and Electrode Inks

Room temperature vulcanizing (RTV) silicone LOCTITE SI 595 CL (Henkel AG & Co.) is used as the dielectric ink. For the electrode ink, silicone thinner (WS‐DA‐G20, Shanghai Wellion) is weighted into ELASTOSIL LR 3162 Part A and Part B (Wacker) separately at a content of 28.6 wt.%, mixed in a planetary mixer (ARV‐310, Thinky) at 2,000 rpm for 6 min. After being mixed with silicone thinner, ELASTOSIL LR 3162 Part A and Part B are mixed (2,000 rpm, 2min) at a 1:1 weight ratio.

### Rheological Characterization of Dielectric and Electrode Inks

Rheological characterization of the dielectric and electrode inks are conducted by using a rotational rheometer (AR‐G2, TA Instrument) with 20mm diameter steel plate geometry. The apparent viscosity is measured as a function of the shear rate by steady‐state flow tests with a logarithmic sweep of shear rate (0.01–100 s^−1^). The shear storage modulus (G′) and loss modulus (G″) are measured as a function of the shear stress via oscillation tests with a logarithmic sweep of shear stress (1–1000 Pa) at a shear frequency of 1 Hz and an oscillatory strain of 0.02. The shear yield stress for each sample is identified as the shear stress at which the shear and loss moduli are the same.

### Procedures for Multimaterial 3D Printing

The prepared dielectric and electrode inks are loaded into 30 cc syringe barrels and centrifuged at 2,000 rpm for 4 min to remove any trapped air bubbles. The inks are then mounted to the customized independent dual extrusion 3D printer (Figure S1, Supporting Information). The pressure supplied to the syringes is controlled by Ultimus V pressure regulator (Nordson EFD) or electro‐pneumatic regulator ITV 2050–312L (SMC Co.) which are connected to the 3D printer motherboard (Skr pro V1.2, Bigreetech). The nozzles with an inner diameter of 210 µm (Smoothflow Tapered Tip, Nordson EFD) are used. All 3D models are designed by Solidworks (Dassault Systèmes). The 3D parts are assembled in commercial software Simplify3D and then converted into the corresponding G‐code. Teflon films are used to prevent the dielectric ink from sticking on the printing platform. The printed fiber diameter is measured by using a digital microscope system (Leica DMS300).

### 3D Printing of a Soft Capacitive Sensor

The printing process of the soft capacitive sensor is illustrated in Figure S22 (Supporting Information). First, a dielectric layer is printed as the substrate. Next, one conductive layer is employed as the bottom electrode. Then the tilted microstructure layer, by using the dielectric silicone ink, is used as the intermediate dielectric elastomer, without any need of supporting materials. It should be emphasized that the design of this middle layer can play a dominant effect on the performance of the soft sensor. See Figure S2 and Table S1(Supporting Information) for a comparison of several designs of intermediate dielectric elastomer. After the printing process, the sensor is cured at a high temperature of 110 °C for ≈0.5 h. For the electrical connection, the sensor leads are 3D‐printed directly (Figure S22, Supporting Information) to allow for easy insertion of the cables. We use conductive silicone to ensure good contact between the sensor leads and the cables. Finally, we apply heat shrink tubing around the sensor leads and cables to create a mechanically robust connection.

### Properties of Soft Capacitive Sensors

The capacitance is measured by using an inductance capacitance and resistance (LCR) meter (E4980AL, KEYSIGHT) under 10 kHz and 1 V via a customized Matlab program. The initial capacitance of the sensor is 2.4 pF, and the capacitance increases to 37.9 pF at a pressure of 5 MPa. Some cost‐effective capacitance‐to‐digital converter chips are capable of detecting such large capacitance changes, such as CN0552 (Analog Devices, America), PCAP02 (ScioSense, Netherlands), etc. A high‐precision Universal Testing Machine (AGS‐X 500N, Shimadzu) is used to control the pressure applied to the sensor. The response time is measured by quickly loading a pressure of 5 kPa on the sensor. Cyclic compression tests of the sensor are carried out by compressing the sensor 20000 times with a cyclic pressure of 420 kPa. The sensor's detection limit is investigated by repeatedly loading a small piece of paper (0.85 Pa) and then recording the capacitance response. The resistances of the electrode and strain sensor are measured by using Data Acquisition System (DAQ970A, KEYSIGHT). For the insole and soft robotic hand, a capacitance to digital converter (Eval AD7746EBZ, Analog Devices) is used with two 16‐channeled analog multiplexers (CD74HC4067, SparkFun), so that the capacitive sensor array can be read sequentially by only one converter. An I2C protocol connection is established between the Arduino Mega 2560 and the Eval AD7746EBZ for the transfer of capacitance data. The capacitance measurement circuit is programmed in MATLAB.

### Sensing Mechanism with High Loading Pressure

The sensor's sensitivity can be inversely proportional to its compressive stiffness. When the pressure is high, it is reasonable to assume that the dielectric layer has become solid. Considering the incompressibility of soft materials, the initial area *A*_0_ and initial thickness *d*_0_ of the dielectric layer are related as follows:

(1) $$A_{0}\;d_{0}=\left(A_{0}-\Delta A\right)\;\left(d_{0}-\Delta d\right)$$

In the deformed state, the relationship can be expressed as:

(2) $$C_{0}+\Delta C=\frac{\varepsilon_{0}\varepsilon_{r}\left(A+\Delta A\right)}{d_{0}-\Delta d}\;$$

where ε_0_ denotes the vacuum permittivity, ε_*s*_ is the relative permittivity of dielectric, and *C*_0_ is initial capacitive of the sensor. So from Equation (2) we have:

(3) $$\frac{\Delta C}{C_{0}}=\frac{\frac{\varepsilon_{0}\varepsilon_{r}\left(A+\Delta A\right)}{d_{0}-\Delta d}-C_{0}}{C_{0}}={\left(\frac{d_{0}}{d_{0}-\Delta d}\right)}^{2}\;-1$$

For simplicity, assuming a linear material, the stress‐strain relationship is given by:

(4) σ=Eε

where 𝐸 is the compression modulus, 𝜎 is the applied compression stress, 𝜀 is the strain. Consequently,

(5) $$\Delta p=E\frac{\Delta d}{d_{0}}$$

So that the sensitivity is:

(6) $$\frac{\frac{\Delta C}{C_{0}}}{\Delta P}=\left({\left(\frac{d_{0}}{d_{0}-\Delta d}\right)}^{2}-1\right)/\Delta p=\left({\left(\frac{1}{1-\frac{\Delta P}{E}}\right)}^{2}-1\right)/\Delta p$$

Therefore, under high pressure, the increase in capacitance of the sensor is not only due to the reduced spacing between the electrodes but also results from the enlarged electrode surface area, as observed in Movie S2 (Supporting Information).

### Stability Testing

The sensor is attached to the base of a three‐axis motorized linear stage by using a silicone rubber adhesive (Sil‐Poxy, Smooth‐On). A square shape indenter (10mm) is connected to the motion stage. Sandpaper of 800 mesh is attached to the indenter to increase the friction force. A 6‐Axis force and torque sensor (Nano 17, ATI) is used to measure the normal and shear forces. For the rubbing tests, the pressure applied to each sensor is 160 kPa, and the test speed is 2 mm s^−1^, in order to realize a reciprocating displacement of 8 mm for 100,000 cycles.

### Mechanical Characterization

Samples of the dielectric and conductive elastomers are printed in a dog‐bone shape and subjected to tensile testing according to ASTM D412C. The tests are performed using a Universal Testing Machine (AGS‐X 500N, Shimadzu) equipped with pneumatic grippers, operating at a testing speed of 500 mm min^−1^. To measure the interfacial toughness, each sample, of length 30mm and width 10 mm, is prepared and subjected to a standard 180° peel test (Figure S23, Supporting Information) by using a Universal Testing Machine. The peeling speed is controlled at 50 mm min^−1^. The sensors are attached to a Bopp sheet by using a silicon rubber adhesive (Sil‐Poxy, Smooth‐On) as a stiff backing.

### Printing of Soft Insole

The insole 3D model consists of two parts, one for the conductive silicone material, and the other for the dielectric one. We print the insole embedded with the sensor array by following the sequences as shown in Figure S24 (Supporting Information). Each layer thickness is set to be 200µm. The sensors are arranged in a 2 × 8 array, and are connected via 10 wires to a multiplexer for measuring each capacitance across the array.

### Printing of Soft Robotic Hand

The finger model consists of six parts, as shown in Figure 5c. The soft finger with multiple sensors is printed by following the sequences as illustrated in Figure S25 (Supporting Information). The length of the thumb is 63 mm, shorter than another four fingers. The printing parameters are listed in Table S4 (Supporting Information). The palm is 3D‐printed with the Polylactic Acid (PLA) by using a Fused Deposition Modeling (FDM) 3D printer (Ultimaker S2, Netherlands). The soft finger and palm are assembled by using silicone rubber adhesive and bolts.

## Conflict of Interest

The authors declare no conflict of interest.

## Author Contributions

J.Z. conceived the idea and directed the study. F.X. conceived the idea, designed the experiments, and conducted related data analysis and interpretation. F.X. and Z.W. builted the multimaterial 3D printer and wrote the Matlab code for capacitance measurement circuit. Z.X., H.W., and J.L. advised on the design and fabrication of the sensors. Z.X. and H.W. also participated in the experiments. F.X. and J.Z. wrote the manuscript. F.X., J.Z., and J.L. revised the manuscript. All authors discussed the results.

## Supporting information



Supporting Information

Supplemental Movie 1

Supplemental Movie 2

Supplemental Movie 3

Supplemental Movie 4

Supplemental Movie 5

Supplemental Movie 6

Supplemental Movie 7

## Data Availability

The data that support the findings of this study are available from the corresponding author upon reasonable request.
