# Supplementary material for: Fully 3D‐Printed Soft Capacitive Sensor of High Toughness and Large Measurement Range
Source: Adv Sci (Weinh). 2025 Jan 7;12(8):2410284. doi: 10.1002/advs.202410284 (PMC11848610; doi:10.1002/advs.202410284)
Supplement: Supplementary file 1 — Supporting Information [file ADVS-12-2410284-s005.pdf]

ADVANCED SCIENCE
Open Access

Supporting Information

for *Adv. Sci.*, DOI 10.1002/advs.202410284

Fully 3D-Printed Soft Capacitive Sensor of High Toughness and Large Measurement Range

*Fei Xiao, Zhuoheng Wei, Zhipeng Xu, Hao Wang, Jisen Li and Jian Zhu**

Supporting Information

Fully 3D-printed Soft Capacitive Sensor of High Toughness and Large Measurement Range

*Fei Xiao, Zhuoheng Wei, Zhipeng Xu, Hao Wang, Jisen Li, Jian Zhu**

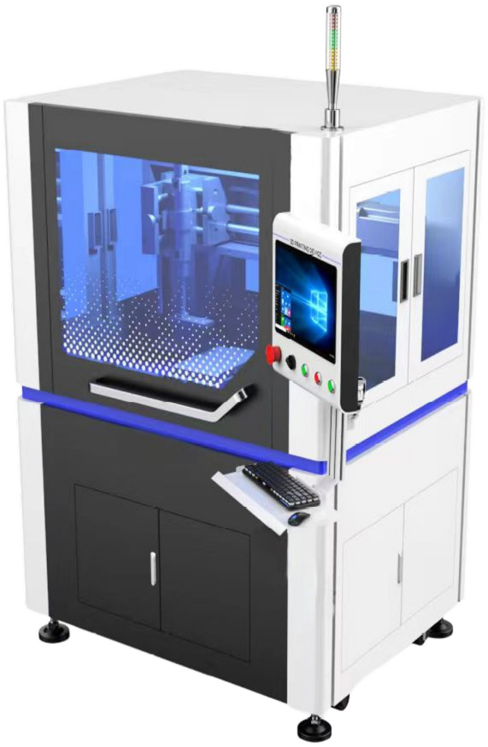

Figure S1. The customized DIW 3D printer.

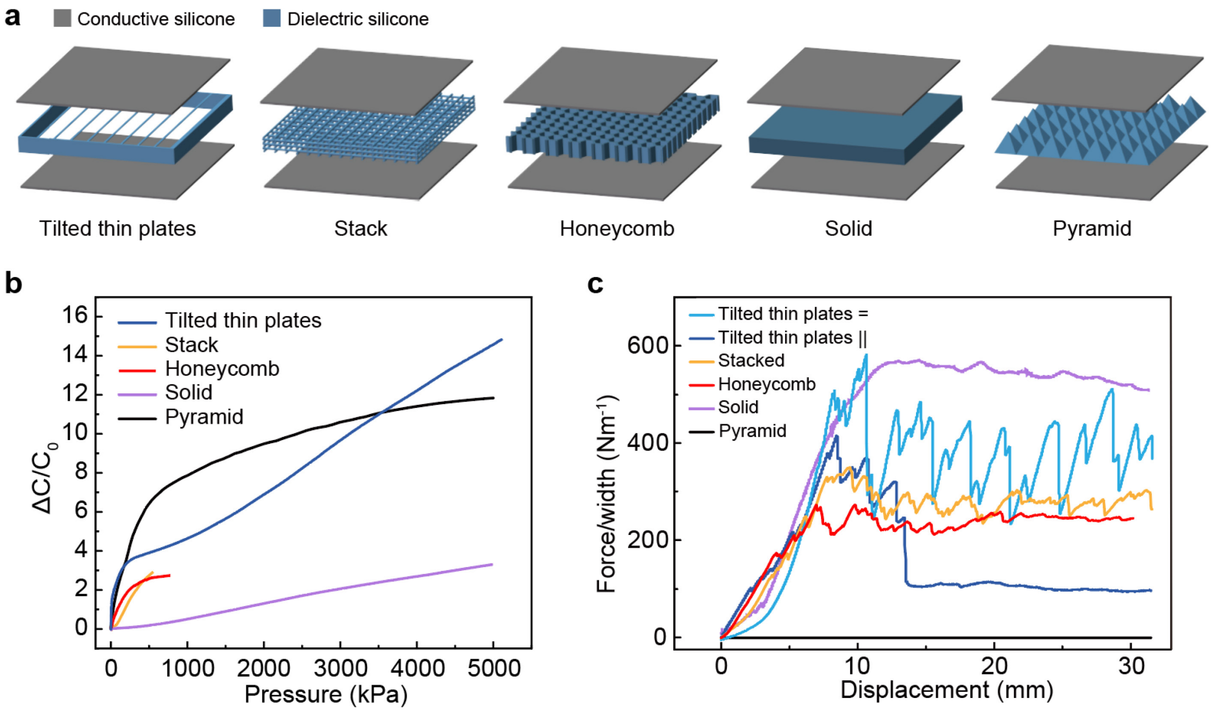

Figure S2. a) Five designs of dielectric structure for 3D-printed soft capacitive sensors. For the pyramid type, the electrode and dielectric are printed separately. b) Capacitance change as a function of applied pressure for the sensors with five designs of dielectric structure. c) Peel force as a function of displacement for the sensors with five designs of dielectric structure.

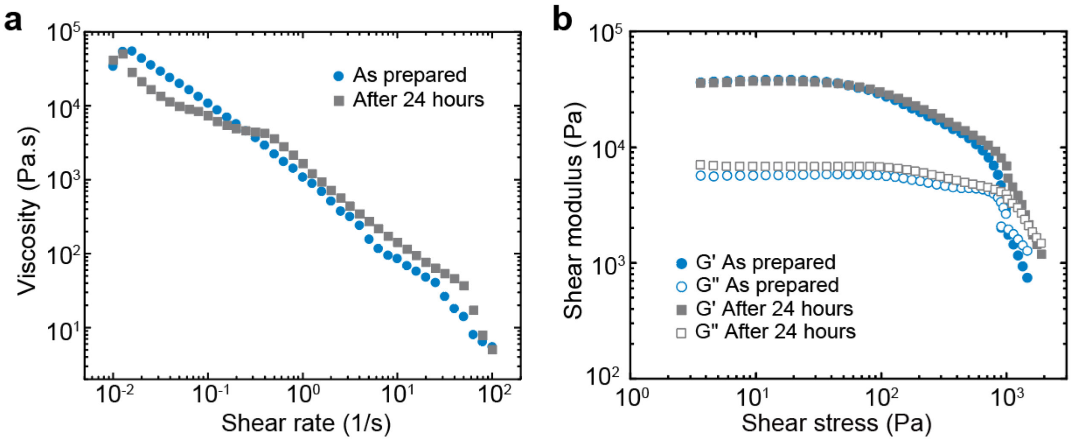

Figure S3. Stability of the electrode inks during storage at room temperature (25°C) over 24 hours. a) Apparent viscosity as a function of applied shear rate. b) Storage modulus as a function of applied shear stress. Nearly no change in the rheological properties is observed, indicating good printability of the inks over a long time.

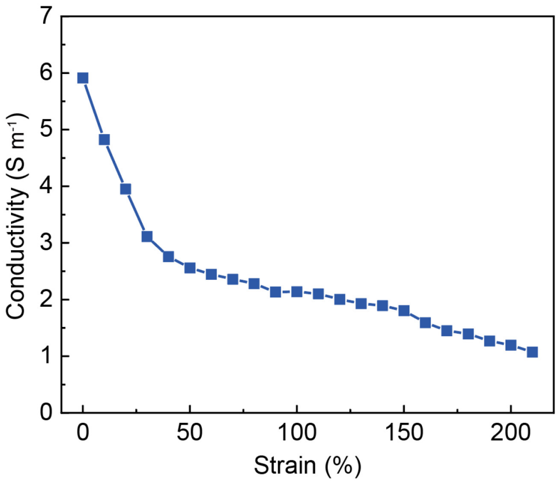

Figure S4. Electrical conductivity as a function of strain for cured electrode inks.

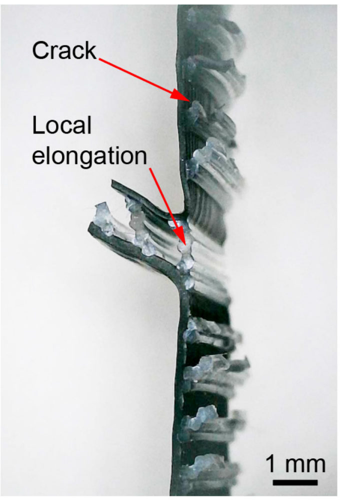

Figure S5. Microscope image of the sensor under 180° peel test.

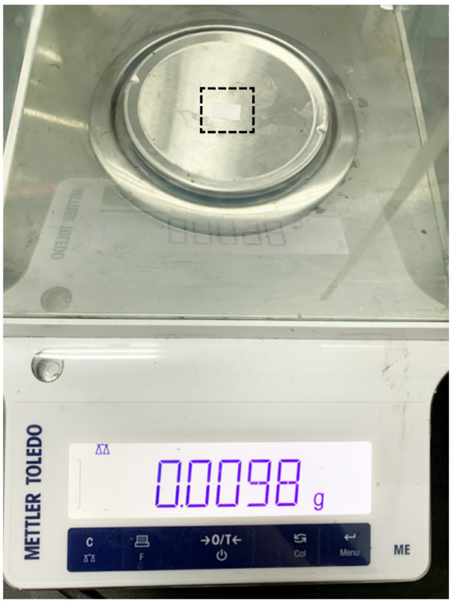

Figure S6. The sensor can detect a piece of paper with a small pressure of 0.85 Pa. A piece of paper with a weight of 0.0098 g, and an area of 13 mm × 8.8 mm, produces a pressure of 0.85 Pa.

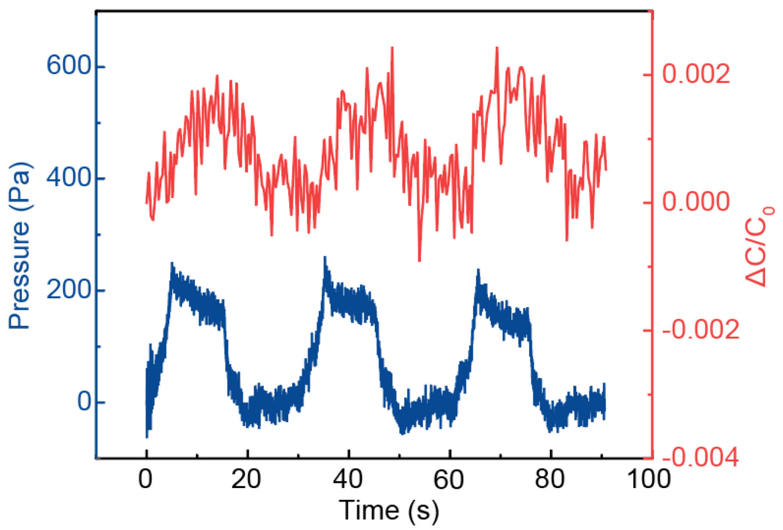

Figure S7. The detection limit of the 3D printed sensor with a solid dielectric layer.

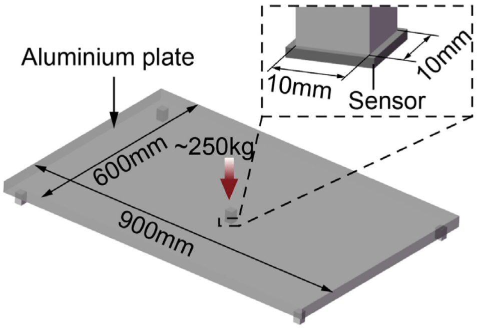

Figure S8. Experimental setup of an aluminum plate, which is supported by five dots each with an area of 1 cm^2. Four adults, with a total weight of approximately 250 kg, stand on the aluminum plate, resulting in a pressure of 5000 kPa on the sensor.

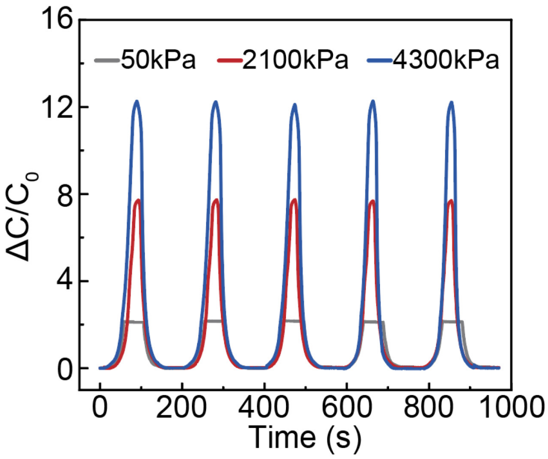

Figure S9. Capacitance change of the sensor under cyclic loading with an amplitude of 50 kPa, 2100 kPa, or 4300 kPa, respectively. The sensor exhibits excellent repeatability.

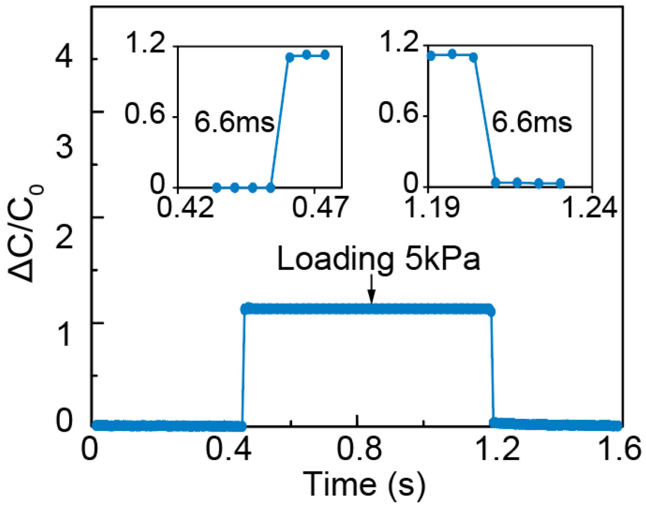

Figure S10. Response and relaxation time of the sensor.

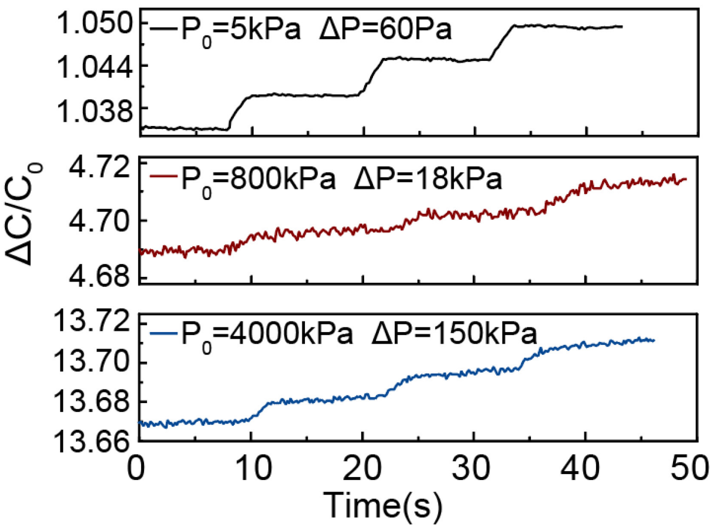

Figure S11. Detection of sequential subtle pressures with three initial loading pressure levels (i.e., 5 kPa, 800 kPa, and 4000 kPa).

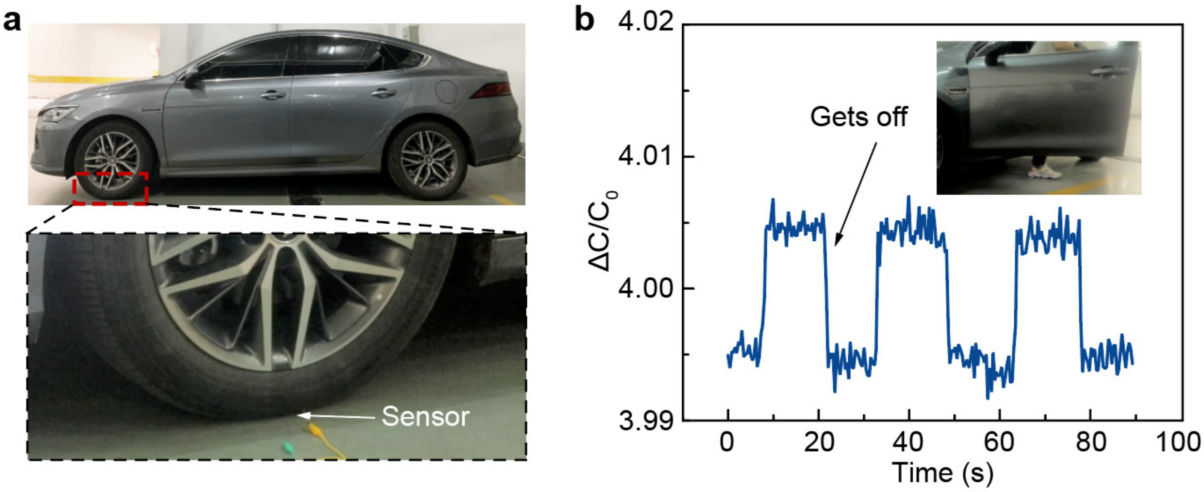

Figure S12. a) Experimental setup of the sensor attached to a tire tread. b) Detection of a 65 kg male getting on and off the car three times. The car weighs 2000 kg, the wheel width is 22.5 cm, and the contact area between each tire and the ground is 22.5 × 8 = 180 cm^2. The total contact area between the 4 tires and the ground is 180 × 4 = 720 cm^2. Consequently, the pressure of car tires on the ground is about 280 kPa. When the human weight is 60 kg, the increased pressure on the car is 8.3 kPa.

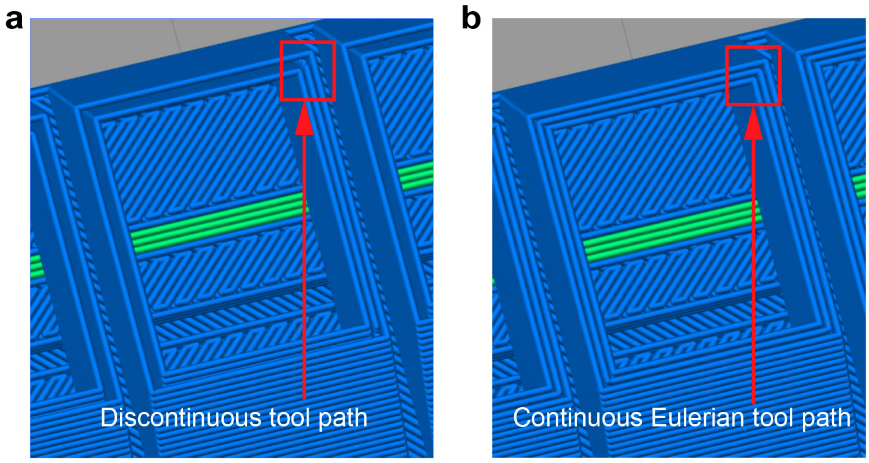

Figure S13. Comparison of soft pneumatic actuators printed with different tool paths. a) Discontinuous tool path, which may decrease printing quality and even lead to printing failure. b) Continuous Eulerian tool path. The printing nozzle is traced through the whole pattern in a single continuous path without interruption. The wall thickness is a multiple of the line width.

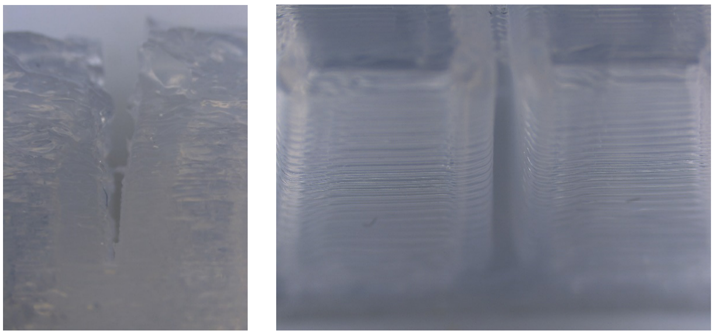

Figure S14. After optimizing the printing paths, the surface of the fingers is smooth.

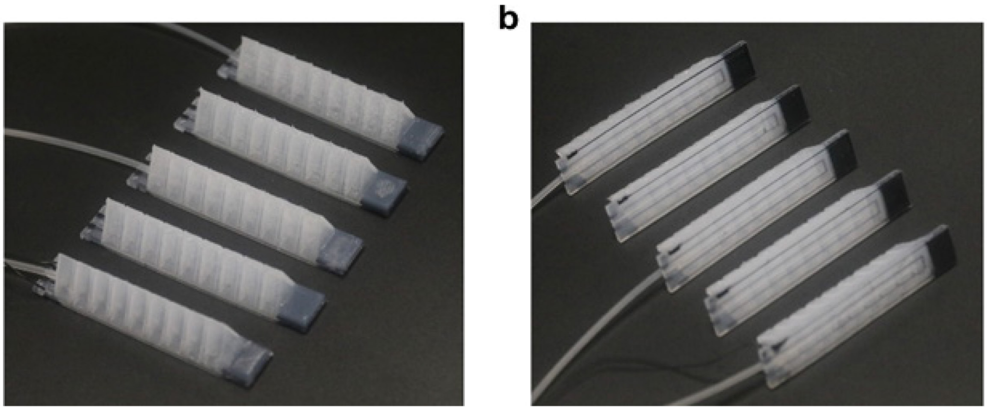

Figure S15. Snapshots of five printed soft fingers. a) Each sensor exhibits excellent print quality with smooth surfaces and without air leakage. b) The two sensors with their bottoms visible, indicating successful printing and proper alignment.

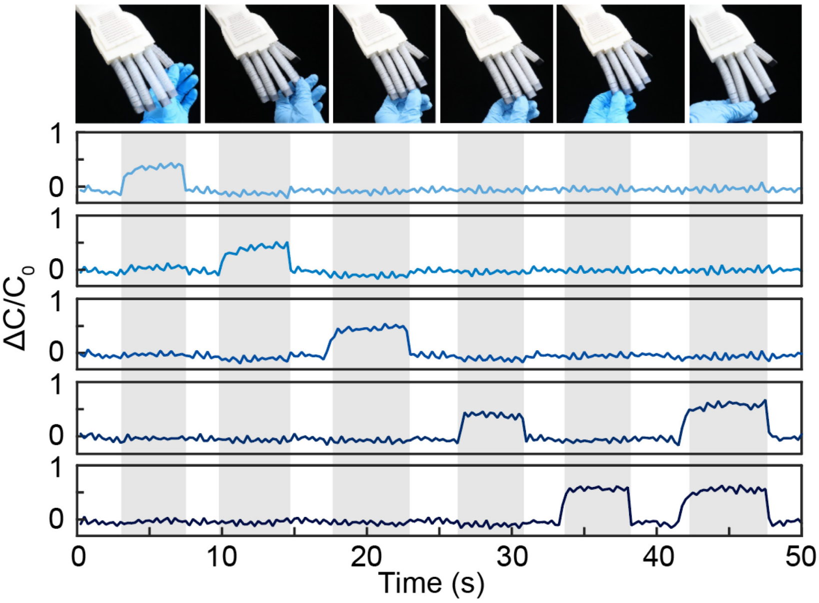

Figure S16. Demonstration of touch sensation of individual fingers or multiple fingers when compressed.

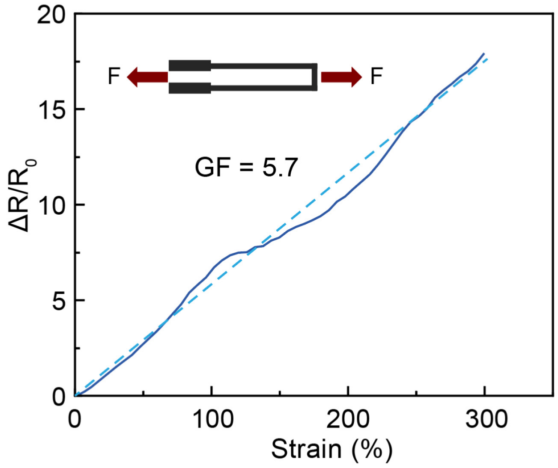

Figure S17. Normalized change in resistance as a function of strain for the flat electrode, demonstrating a constant gauge factor of 5.7 in the strain range of 0–300%.

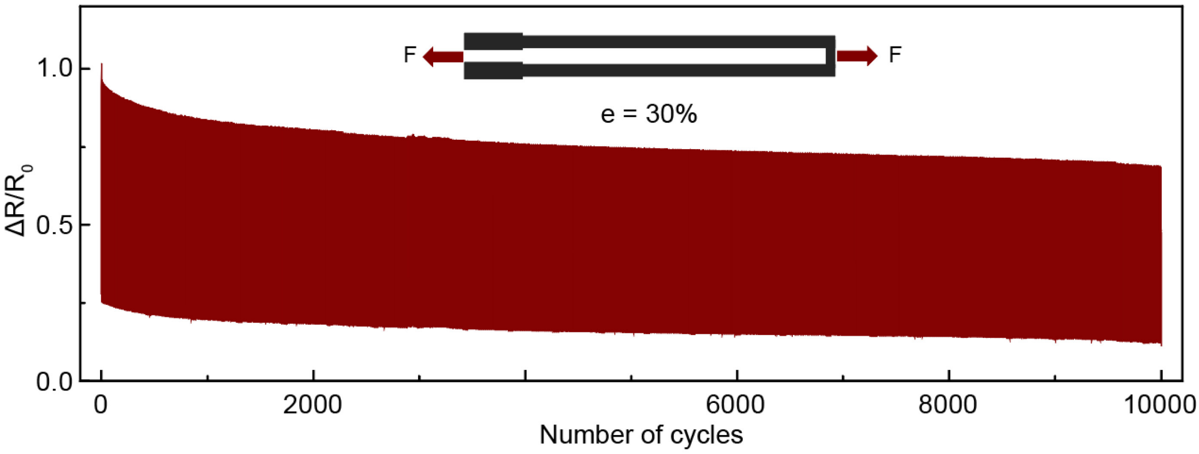

Figure S18. Normalized change in resistance of the silicone electrode stretched to a strain of 30% over 10,000 cycles.

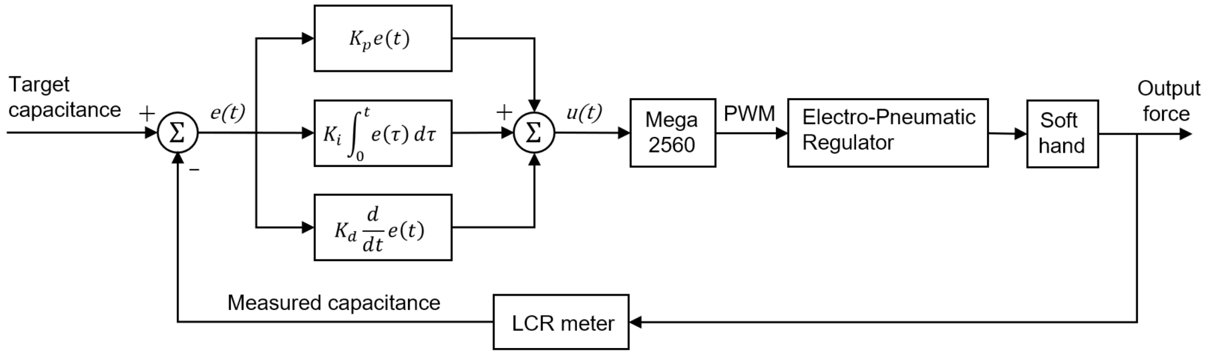

Figure S19. Schematic of closed-loop PID force controller.

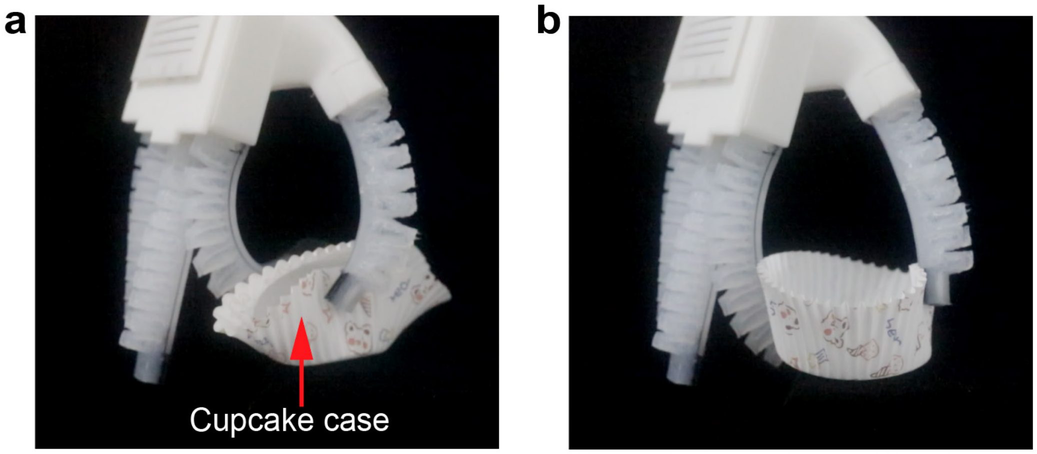

Figure S20. Grasp a cupcake case using a) open-loop or b) closed-loop PID force controller, respectively.

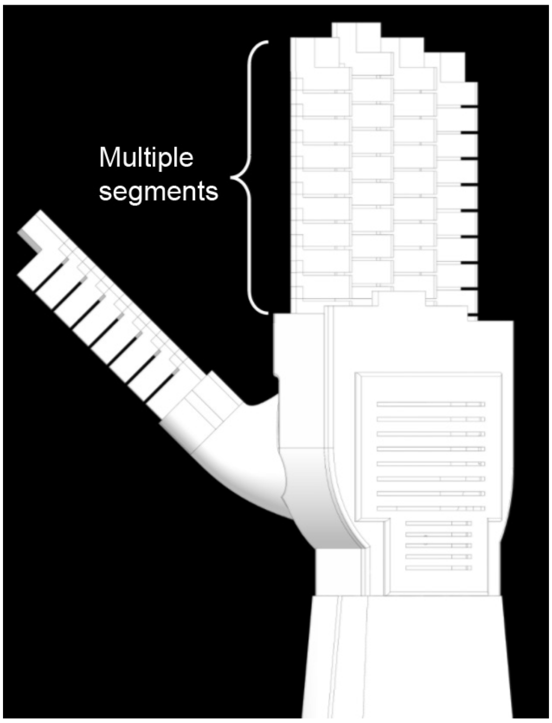

Figure S21. Each finger is divided into multiple segments, and each segment is assumed to have the same bending curvature in the simulation.

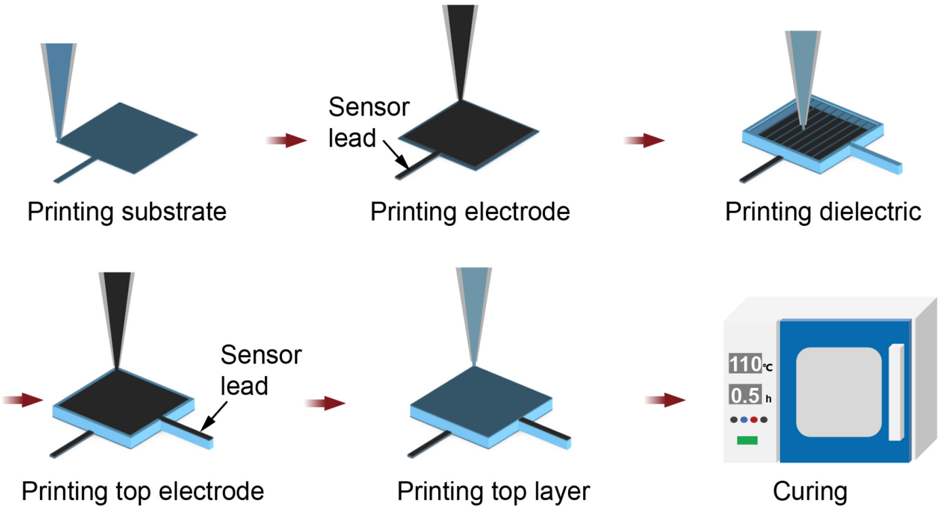

Figure S22. Schematic illustration of the multimaterial DIW process for fabricating a soft capacitive pressure sensor. The electrode and dielectric inks are printed sequentially. After printing, the sensor is cured at a high temperature of 110°C for 30 minutes. The sensor leads are 3D-printed directly to allow for easy insertion of the cables.

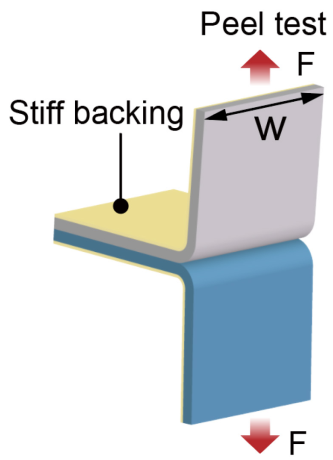

Figure S23. Schematic of standard 180° peel test for interfacial toughness measurement. The interfacial toughness is determined by dividing two times the plateau force by the width of the sensor sample.

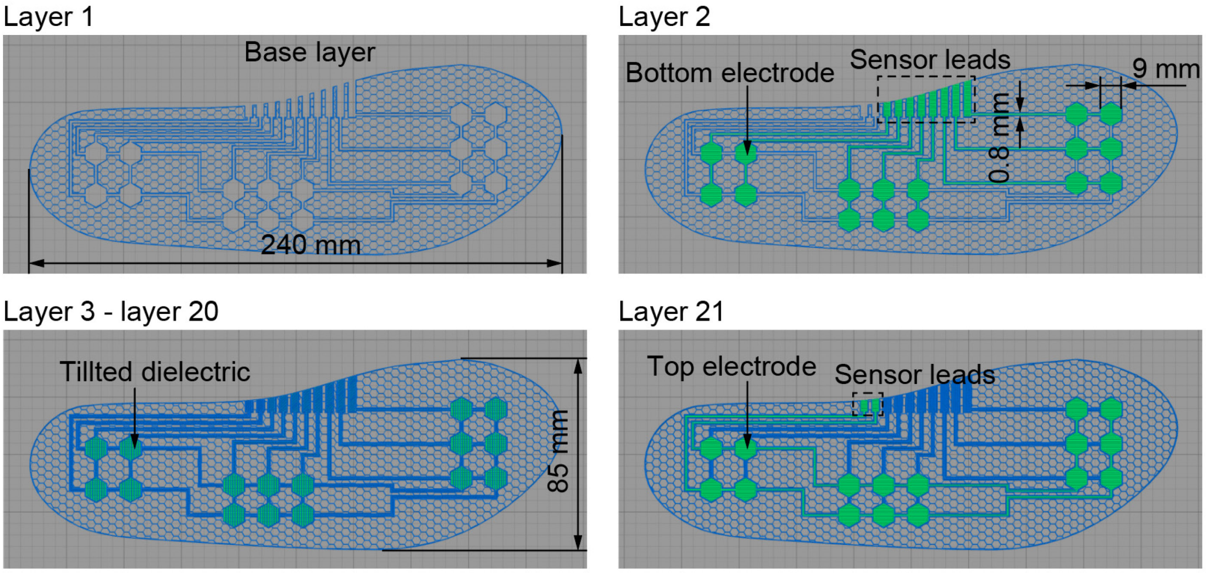

Figure S24. Printing procedures and geometric parameters of the soft insole embedded with the sensor array.

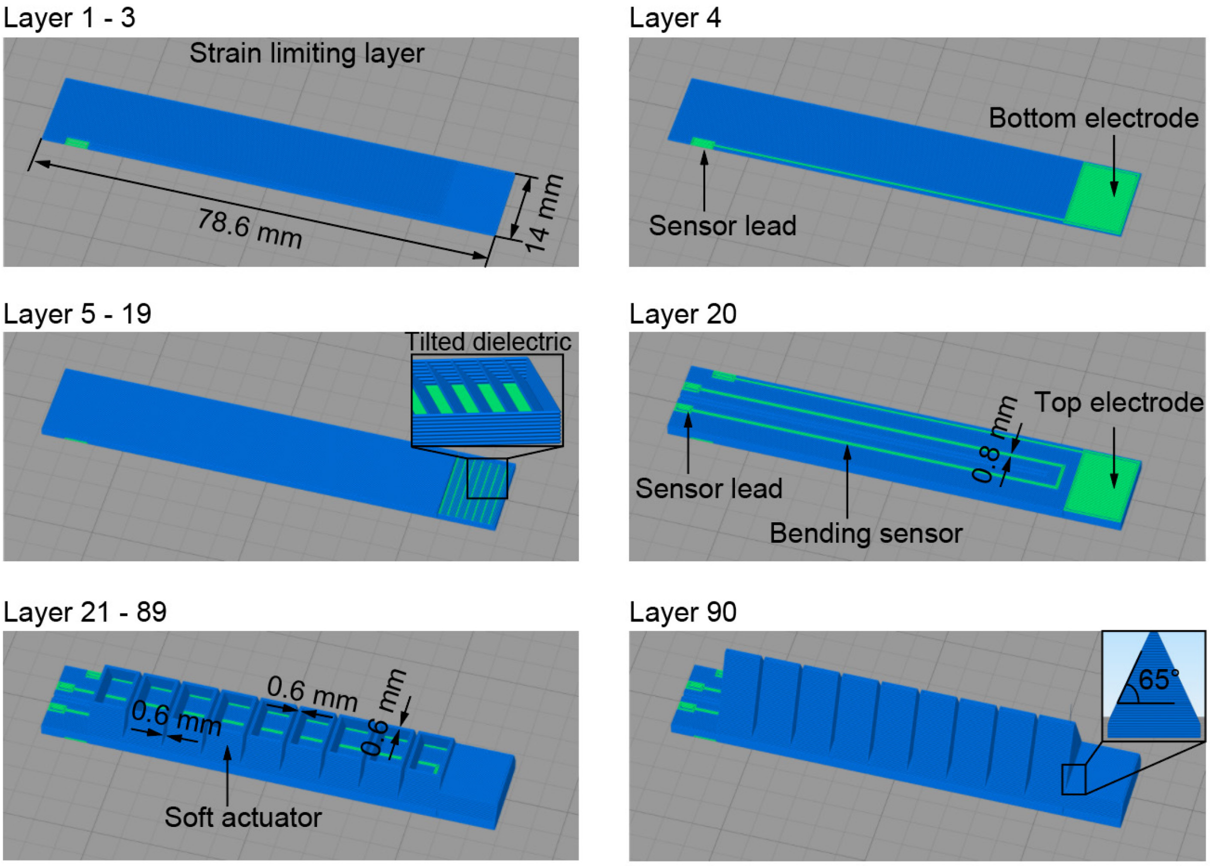

Figure S25. Printing procedures and detailed geometric parameters of the soft finger.

Table S1. Comparison in performance of soft capacitive sensors with various dielectric structures.

Dielectric structure	Measurement range (kPa)	Interfacial toughness ($J \cdot m^{-2}$)	Sensitivity (kPa^{-1})
Tilted thin-plates	0.00085-5000	645 (transverse)<br>339 (longitudinal)	0.22
Stack	0.005-550	512	0.037
Honeycomb	0.003-760	390	0.04
Solid	0.2-5000	1039	0.0007
Pyramid	0.0005-5000	0	0.3

Table S2. Comparison in measurement range between our 3D-printed sensor with soft capacitive sensors in the literature.

Measurement range	Detection limit (Pa)	Maximum pressure (kPa)	Ref.
Neither sense low nor high pressure (3D printing)	12	2	[34]
	11	14	[39]
	9	4	[40]
	5	550	[43]
	20	10	[36]
	9	280	[56]
Neither sense low nor high pressure	2	200	[57]
	4	25	[58]
	3	10	[59]
	5	30	[60]
Only low pressure	0.21	40	[7]
	0.35	450	[12]
	0.7	10	[21]
	0.07	50	[11]
	0.2	21	[22]
Only high pressure	1000	1080	[25]
	100000	1300	[24]
	2000	1700	[26]
	500	4500	[27]
	50	1000	[6]
Both	1	1000	[61]
Both	0.85	5000	This work

Table S3. Comparison in measurement range between our 3D-printed sensor and iontronic sensors in the literature.

Measurement range	Detection limit (Pa)	Maximum pressure (kPa)	Ref.
(3d printing) Neither sense low nor high pressure	26	70	[35]
	N/A	5	[41]
Neither sense low nor high pressure	1000	750	[75]
	35	330	[76]
	1.12	30	[77]
Only low pressure	0.08	360	[53]
	0.2	300	[78]
	0.4	120	[79]
Only high pressure	1.7	1000	[80]
	5	1000	[81]
	300	2500	[23]
	13	2000	[82]
	7.2	3360	[83]
Both	0.36	3000	[84]
	0.25	4000	[18]
Both	0.85Pa	5000	This work

Table S4. Design and control parameters for printing a soft finger combining soft actuators and soft sensors.

Designed vertical wall thickness for the pneumatic actuator	0.6
The layer of vertical walls for the pneumatic actuator	3
Line width	0.2um
Layer height	0.2um
Nozzle diameter	0.2um
Printing speed and pressure (Dielectric silicone)	10mm/s, 300kpa
Printing speed and pressure (Conductive silicone)	10mm/s, 320kpa
Printing speed and pressure (Tilted dielectric)	10mm/s, 300kpa for α=0°<br>7mm/s, 300kpa for α=30°<br>6mm/s, 300kpa for α=45°<br>4mm/s, 300kpa for α=60°
Printing speed and pressure (Pneumatic actuator)	10mm/s, 340kpa
Infill density	100%
Top solid layers	0
Bottom solid layers	0
Perimeter shells	1
External Thin Wall Type	Allow single extrusion wall
Internal Thin Wall Type	Allow gap fill
Allowed perimeter overlap	0%

Supplementary Movies Captions

Movie S1: Measurement of the detection limit of the soft capacitive sensor. The sensor can sense a piece of paper (~0.85 Pa).

Movie S2: Changes in capacitance and strain of the 3D-printed sensor when the sensor is loaded with 0 to 5000 kPa. The sensor can achieve a large measurement range.

Movie S3: Real-time monitoring of plantar pressure distribution using the 3D-printed intelligent insole.

Movie S4: Demonstration of tactile sensation of the 3D-printed fingers. The five tactile sensors can detect applied forces and decoupling them from each other.

Movie S5: Comparison of an open-loop controller and a closed-loop controller to grasp a cupcake case. With the soft tactile sensors, the soft robotic hand can achieve force-controlled grasping of a cupcake case.

Movie S6: Controlling a virtual soft robotic hand in Unity using the bending sensors in the soft robotic hand. The virtual hand can repeat the posture of our soft robotic hand in a real-time manner.

Movie S7: Grabbing objects of various shapes utilizing both the tactile and bending sensors. The soft robotic hand can grab these objects effectively, based on the fusion of the tactile and bending sensors.

References

[75] B. Nie, R. Li, J. Cao, J. D. Brandt, T. Pan, *Advanced Materials* **2015**, *27* (39), 6055.
[76] Z. Shen, X. Zhu, C. Majidi, G. Gu, *Advanced Materials* **2021**, *33* (38), 2102069.
[77] A. Chhetry, J. Kim, H. Yoon, J. Y. Park, *ACS Applied Materials & Interfaces* **2019**, *11* (3), 3438.
[78] Z. Ding, W. Li, W. Wang, Z. Zhao, Y. Zhu, B. Hou, L. Zhu, M. Chen, L. Che, *Advanced Science* **2024**, *11* (24), 2309407.
[79] Q. Liu, Z. Liu, C. Li, K. Xie, P. Zhu, B. Shao, J. Zhang, J. Yang, J. Zhang, Q. Wang, C. F. Guo, *Advanced Science* **2020**, *7* (10), 2000348.
[80] P. Li, L. Xie, M. Su, P. Wang, W. Yuan, C. Dong, J. Yang, *Nano Energy* **2022**, *101*, 107571.
[81] H. Lee, R. Sharma, S. Park, Z. Bao, H. Moon, S. Yoo, *Advanced Functional Materials* **2024**, *34* (7), 2302633.
[82] Y. Xiao, Y. Duan, N. Li, L. Wu, B. Meng, F. Tan, Y. Lou, H. Wang, W. Zhang, Z. Peng, *ACS Sensors* **2021**, *6* (5), 1785.
[83] Z. Chen, Y. Zhang, B. Zhu, Y. Wu, X. Du, L. Lin, D. Wu, *ACS Applied Materials & Interfaces* **2022**, *14* (17), 19672.
[84] R. Yang, A. Dutta, B. Li, N. Tiwari, W. Zhang, Z. Niu, Y. Gao, D. Erdely, X. Xin, T. Li, H. Cheng, *Nature Communications* **2023**, *14* (1), 2907.
